# Equity and representation in oncology clinical evidence for FDA-approved treatments and the impact of COVID-19

**DOI:** 10.3389/fonc.2026.1666497

**Published:** 2026-05-01

**Authors:** Hao Cheng, Jun Li, Ningying Mao

**Affiliations:** School of International Pharmaceutical Business, China Pharmaceutical University, Nanjing, China

**Keywords:** FDA approvals, diversity, equity, oncology, clinical trials

## Abstract

**Background:**

Pivotal clinical trials form the evidentiary foundation for FDA oncology drug approvals, yet persistent disparities in participant demographics may limit the generalizability and equity of cancer care. This study aimed to quantify demographic disparities in pivotal trials that supported FDA oncology drug approvals, and to identify trial-level characteristics associated with the underrepresentation of women, racial and ethnic minorities, and older adults. We also assessed the impact of COVID-19 on participant representation.

**Methods:**

We systematically identified pivotal trials supporting FDA oncology drug approvals from January 2018 through November 2024 using the FDA Hematology/Oncology Approvals database. Demographic data were extracted for gender, race, ethnicity, and age. SEER 21 data were used to derive population-based estimates. Enrollment incidence ratios (EIRs) were calculated for gender, race, ethnicity, and older adults (EIR <1 indicating underrepresentation). Meta-regression was used to evaluate temporal trends and associations between trial characteristics and representation disparities. Subgroup analyses were conducted for pediatric trials, accelerated approvals, and other trial-level factors.

**Results:**

Among 329 included trials enrolling over 164,000 participants, women, older adults, Black, and Hispanic individuals were consistently underrepresented. Pooled EIRs indicated significant underrepresentation of female (EIR, 0.83; 95% CI, 0.78–0.88), Black (EIR, 0.26; 95% CI, 0.22–0.30), Hispanic (EIR, 0.50; 95% CI, 0.44–0.57), and older participants (EIR, 0.86; 95% CI, 0.83–0.89). Meta-regression showed modest gains in female representation over time (β=0.02, p=0.03), but no significant improvement for Black or Hispanic participants. Older adult representation declined slightly. Trial-level factors associated with underrepresentation included industry sponsorship, use of overall survival as the primary endpoint, and accelerated approval designation. Pandemic-period EIRs were largely similar to non-pandemic EIRs, with lower representation for older adults and White participants.

**Conclusions:**

Despite recent regulatory efforts, demographic disparities persist in pivotal oncology trials supporting FDA approvals. Female, Black, Hispanic, and older adults remain underrepresented, limiting the applicability of trial findings to real-world cancer populations. Greater efforts are needed to design inclusive trials, enforce diversity mandates, and ensure equitable evidence generation in oncology drug development.

## Introduction

Pivotal clinical trials serve as the primary source of evidence for oncology drug approvals by the US Food and Drug Administration (FDA), forming the basis for regulatory decisions and supporting labeling claims (1). The results of these trials also influence clinical guidelines, reimbursement policies, and the integration of new therapies into routine practice (2, 3). Given their central role in shaping cancer care, it is essential that trial populations reflect the demographics of those affected by the disease. When certain groups are underrepresented, concerns arise about the applicability of the findings and the potential for disparities in treatment outcomes across real-world populations (4).

Despite longstanding awareness of these issues, disparities in trial representation remain widespread. In trials that supported FDA oncology drug approvals from 2008 to 2018, Kalathoor et al. (5) found that women accounted for just 40.7% of participants, and Loree et al. (6) showed that Black and Hispanic patients made up only 3.1% and 6.1% of participants, respectively, far below their proportionate cancer burden in the US population. Although older adults account for the majority of cancer cases, they remain significantly underrepresented in oncology clinical trials (7).

In recent years, several federal policies have sought to address these gaps. National Institutes of Health (NIH) requires justification of enrollment goals by sex, race, and ethnicity, requiring researchers to justify enrollment goals for sex, race, and ethnicity in NIH-funded studies in 2018 (NOT-OD-18-014) (8). FDA’s 2020 guidance recommends broadening eligibility, decentralizing access, and engaging underrepresented groups (9). Most recently, the 2022 Food and Drug Omnibus Reform Act (FDORA) authorized the FDA to require Diversity Action Plans (DAP) for pivotal trials, including targets by sex, race, ethnicity, and age (10).

Despite these efforts, it remains unclear whether participant representation has improved since 2018 in pivotal oncology trials that support FDA approvals in the era of precision medicine (11). As targeted therapies and immunotherapies increasingly rely on molecular subtypes, demographic diversity is essential for both equity and scientific rigor (12). To address this gap, we analyzed pivotal clinical trials supporting FDA approvals of oncology drugs from 2018 to 2024. We assessed the representation of women, racial and ethnic minorities, and older adults, and examined whether diversity varied by trial characteristics. We also evaluated whether participant representation differed during the COVID-19 pandemic period. Findings from this study may inform future regulatory strategies to improve inclusion in cancer research.

## Method

### Search strategy and study selection

We reviewed all oncology drug approvals by the US Food and Drug Administration (FDA) from January 2018 to November 2024, using the FDA Hematology/Oncology Approvals database, which contains comprehensive records of new oncology drug approvals in the US. Two reviewers (H.C. and J.L.) independently screened all records for eligibility according to predefined inclusion and exclusion criteria. Discrepancies were resolved through discussion until consensus was reached. Supporting clinical trials were identified via PubMed and ClinicalTrials.gov using NCT numbers, drug names, and approved indications.

Trials were included if they evaluated solid or hematologic malignancies and directly supported new FDA oncology drug approvals, ensuring that the analysis captured studies with regulatory relevance. Device-based approvals were excluded because they follow distinct regulatory pathways and typically lack standardized demographic reporting comparable to drug trials. Label extensions without new clinical data and approvals referring only to previously published trials without additional enrollment were also excluded to avoid duplicate counting of the same pivotal trials Studies with unavailable primary reports or missing demographic information were further excluded to maintain data completeness and comparability across trials. These criteria ensured that each pivotal trial was analyzed only once and that the dataset reflected unique, regulatory-grade evidence supporting oncology drug approvals.

### Data extraction and reporting

Two reviewers (H.C. and J.L.) extracted trial characteristics, including the proportions of male and female participants, age distribution, and racial and ethnic representation, in accordance with the FDA’s position statement (see eMethods in the Supplement for details). Minor discrepancies between the two reviewers occurred in approximately 8% of extracted items, primarily involving classification of trial design or endpoint type. These were resolved through discussion and mutual agreement. In the rare cases (<2% of items) where consensus could not be achieved, the final decision was made by the senior author (N.M.), who supervised the overall data quality and methodological integrity.

We followed the Preferred Reporting Items for Systematic Reviews and Meta-Analyses (PRISMA) guidelines, adapted for meta-epidemiologic research (13).

### Statistical analysis and outcome measures

#### Measurement of trial indicators

For each trial, we assessed subgroup representation by age, race, ethnicity, and gender. Trial proportions (TPs) were calculated as the number of participants within each subgroup divided by the total number of trial participants. All representation metrics (gender, age, race, and ethnicity) were analyzed in a disease-specific manner according to each trial’s cancer type. Analyses by gender excluded sex-specific cancers (e.g., prostate and breast cancer). Trials reporting participant counts by age group (older patients [≥65 years] vs younger patients [<65 years]) were included in the analysis of age-related disparities. Pediatric trials were excluded only from the older-adult (65 years) analysis, and were retained in analyses of sex, race, and ethnicity when relevant data were reported. To minimize geographic population bias, the primary analysis of racial and ethnic representation excluded indications with higher incidence in Asia than in North America, including liver, gastroesophageal, lung, cervical, and head and neck cancers (6, 14). This restriction was used because many pivotal trials for these indications are globally conducted and may recruit disproportionately from Asian regions. When EIRs are benchmarked against US-only SEER incidence, such geographic recruitment patterns can inflate the estimated EIR for Asian participants and may indirectly lower the relative representation of other groups. We therefore performed a sensitivity analysis including all indications to evaluate the impact of this decision. As expected, re-including all indications yielded a higher pooled EIR for Asian/Pacific Islander participants, consistent with potential geographic recruitment effects in trials for Asia-prevalent cancers. Besides, due to limited reporting and low representation, American Indian and Alaska Native individuals were also excluded from the analysis (15).

#### Acquisition of population-based estimates indicators

We obtained cancer incidence estimates by age (≥65 and <65 years), gender, race, and ethnicity from the National Cancer Institute’s Surveillance, Epidemiology, and End Results (SEER) program database (SEER 21) (16). These data were adjusted to align with indication and median enrollment year of each clinical trial to account for disease- and time-specific differences. SEER was used as the reference source, as its demographic classifications align with FDA guidelines commonly followed in US-based clinical trials (15, 17, 18).

#### Estimation of enrollment incidence ratios and median-age ratios

Enrollment incidence ratios (EIRs) were utilized to assess differences in clinical trial enrollment among age, gender, racial, and ethnic groups. EIR was determined by dividing TP of each subgroup by that subgroup’s disease incidence in SEER:


$$EIR_{subgroup}=\frac{\text{Trial participants in subgroup}/\text{Total trial participants}}{\text{Incident cases in subgroup }(\text{SEER})/\text{Total incident cases }(\text{SEER})}$$

An EIR above 1 signified that a subgroup was overrepresented in the trial relative to its population incidence, whereas an EIR below 1 indicated underrepresentation. To align trial data with population estimates and maintain consistency, individuals categorized as Asian and Pacific Islander were combined. This was done to match SEER race definitions and to improve estimate stability given sparse and inconsistent reporting of Pacific Islander subgroups in trial publications (15). Details regarding methods for estimating uncertainty in EIRs are provided in eMethods.

#### Meta-analysis and meta-regression

Log-transformed EIRs and their standard errors from each trial were synthesized using the inverse variance weighting method. Overall disparities in enrollment were evaluated through a random-effects meta-analysis, with heterogeneity measured by the I² statistic. A random-effects multivariable meta-regression was applied to assess how trial-level characteristics relate to enrollment disparities, using EIRs derived from individual studies (see eMethods for detailed description for multivariable meta-regression model). Additionally, meta-regression was used to evaluate changes in enrollment disparities over time by analyzing the relationship between log-transformed EIRs and FDA approval year of the novel treatment evaluated in each trial. Multicollinearity was evaluated using Variance Inflation Factors, with values <5 indicating no significant collinearity (19). Meta-regression coefficients (β) are estimated on the log(EIR) scale. Exponentiating the coefficient yields an effect size on the EIR scale: exp(β) is the EIR ratio associated with a one-unit increase in a continuous covariate or a category difference relative to the reference group. This corresponds to an approximate percent change of 100*[exp(β)−1]%.

### Sensitivity analyses

To ensure the robustness of our findings and to explore potential sources of heterogeneity, we conducted subgroup analyses based on the following factors: (1) pediatric trial status; (2) FDA approval year; (3) accelerated approval; (4) trial phase; (5) trial design; (6) cancer type; (7) trial size; (8) industry sponsorship; (9) primary endpoint; and (10) sex distribution. Notes for subgroup categories and definitions are provided in **eTable 2**.

We also performed *post hoc* sensitivity analyses to assess racial and ethnic disparities by including all clinical trials regardless of indication. To further reduce the potential influence of globally conducted trials with recruitment in Asia, we additionally assessed representation of Black patients using the EIR based on Black-to-White enrollment ratio in trials compared with Black-to-White ratio reported in the SEER.

We performed an additional pandemic-timing analysis using the trial enrollment midpoint year to classify trials as pandemic (2020–2022) versus non-pandemic; further details are provided in the eMethods.

## Results

A total of 343 pivotal oncology drug trials were initially identified. Fourteen records were excluded for the following reasons: one diagnostic assay approval, six label updates without new trials, and seven studies lacking accessible data. A final total of 329 unique trials were included in the analysis., enrolling 164,726 participants (**Supplementary Figure S1**). Details on approval year, approved therapies, and supporting trials are provided in **eTable 1**. Pediatric trials accounted for 21 (6.4%) studies, accelerated approvals were granted in 61 cases (18.5%), involving 12,974 participants (7.9%). Among the 329 included trials, 208 (63.2%) were phase 3 studies, 225 (68.4%) employed non-randomized designs, and 306 (93.0%) were industry-sponsored. Notably, among the 104 non-randomized pivotal trials identified, only 4 (3.8%) were phase 3 studies. Trial sizes were small (<100 participants) in 41 trials (12.5%), intermediate (100–500) in 166 (50.5%), and large (>500) in 122 (37.1%), the latter accounting for 71.9% of participants. Hematologic malignancies (91 [27.7%]), lung (59 [17.9%]), genitourinary (45 [13.7%]), and digestive system cancers (44 [13.4%]) were most commonly studied. Breast cancer trials (29 [8.8%]) enrolled the largest share of participants (35,788 [21.7%]). Primary endpoints were mostly non-OS, overall survival was used in 72 trials (21.9%). Targeted therapies (122 [37.1%]) and immunotherapies (82 [24.9%]) were the most frequently studied. More details are in **Table 1**.

**Table 1:** Summary of trial and participants characteristics.

Varible	Trial characteristics	Participants characteristics
	no. (percent)	no. (%)
Total	329	164,726
Trial phase		
1	17 (5.17%)	2,523 (1.53%)
2	104 (31.61%)	17,104 (10.38%)
3	208 (63.22%)	145,099 (88.09%)
Approval year		
2018	44 (13.37%)	23,147 (14.05%)
2019	38 (11.55%)	24,774 (15.04%)
2020	60 (18.24%)	24,018 (14.58%)
2021	43 (13.07%)	23,416 (14.22%)
2022	39 (11.85%)	16,741 (10.16%)
2023	44 (13.37%)	26,887 (16.32%)
2024	61 (18.54%)	25,743 (15.63%)
Randomized controlled trial		
Yes	104 (31.61%)	21,940 (13.32%)
No	225 (68.39%)	142,786 (86.68%)
Size of trial, No. of participants		
Small (<100)	41 (12.46%)	2,616 (1.59%)
Intermediate (100-500)	166 (50.46%)	43,699 (26.53%)
Large (>500)	122 (37.08%)	118,411 (71.88%)
Pediatric		
Yes	21 (6.38%)	4,084 (2.48%)
No	308 (93.62%)	160,642 (97.52%)
Clinical setting		
Breast Cancer	29 (8.81%)	35,788 (21.73%)
Childhood Cancers	18 (5.47%)	3,962 (2.41%)
Digestive System Cancers	44 (13.37%)	22,108 (13.42%)
Genitourinary Cancers	45 (13.68%)	32,725 (19.87%)
Hematologic Malignancies	91 (27.66%)	25,873 (15.71%)
Lung and Bronchus	59 (17.93%)	27,624 (16.77%)
Skin Cancers	20 (6.08%)	7,497 (4.55%)
Urinary System Cancers	12 (3.65%)	5,555 (3.37%)
Others (Endocrine, Head and Neck, Nervous System and Respiratory System),	11 (3.34%)	3,594 (2.18%)
Class of treatment regimen		
Targeted therapy	122 (37.08%)	39,951 (24.25%)
Immunotherapy	82 (24.92%)	41,859 (25.41%)
Chemotherapy	11 (3.34%)	3,157 (1.92%)
Cell therapy	16 (4.86%)	2,369 (1.44%)
Antibody-drug conjugate	29 (8.81%)	13,181 (8.00%)
Immunotherapy+Chemotherapy	20 (6.08%)	16,253 (9.87%)
Immunotherapy+Targeted therapy	16 (4.86%)	9,671 (5.87%)
Hormonal therapy	10 (3.04%)	11,292 (6.86%)
Others (Supportive care, Radioligand therapy and Gene therapy)	23 (6.99%)	26,993 (16.39%)
Primary end point		
OS	72 (21.88%)	52,020 (31.58%)
PFS/DFS/RFS/EFS	107 (32.52%)	81,780 (49.65%)
Others (CR/ORR/DOR)	150 (45.59%)	30,926 (18.77%)
Funding source		
Industry	306 (93.01%)	156,572 (95.05%)
Nonindustry	23 (6.99%)	8,154 (4.95%)
Accelerated approval		
Yes	61 (18.54%)	12,974 (7.88%)
No	268 (81.46%)	151,752 (92.12%)

CR, Complete Response; DFS, Disease-Free Survival; DOR, Duration of Response; EFS, Event-Free Survival; ORR, Objective Response Rate; OS, Overall Survival; PFS, Progression-Free Survival; RFS, Relapse-Free Survival.

**Figure 1:**
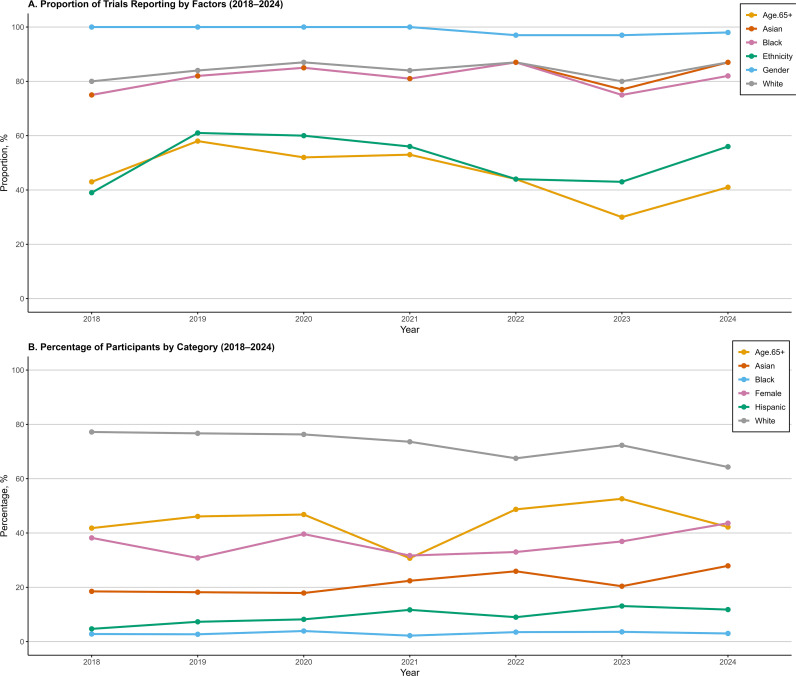
Race and ethnicity reporting and distribution in oncology trials supporting FDA approval of cancer therapies. ^a^ Asian represent Asian or Pacific Islander patients. ^b^ The percentage of Hispanic patients was calculated as their number divided by the sum of Hispanic and Non-Hispanic patients, while percentages for Asian or Pacific Islander, Black, and White patients were calculated as their respective numbers divided by the sum of these three groups. ^c^ **(A)** shows, by year, the proportion of included trials that reported each variable (denominator = all included trials in that year; pediatric trials were excluded for the age ≥65 years reporting metric). ^d^ **(B)** percentages were calculated within the subset of participants from trials that reported the corresponding variable; therefore, denominators differ across race, ethnicity, sex, and age curves and may vary by year. Race percentages were calculated among participants categorized as Asian/Pacific Islander, Black, or White; ethnicity percentages were calculated among participants categorized as Hispanic vs non-Hispanic. Sex percentages were calculated among participants with reported sex (female vs male). Age (≥65 years) percentages were calculated among participants in nonpediatric trials with reported age, consistent with the primary analysis.

Among the 329 trials analyzed, race and ethnicity were reported in 277 (84.2%) and 170 (51.7%) trials, respectively. Excluding sex-specific indications, sex was reported in 260 of 263 trials (98.9%). Of 308 non-pediatric trials, 150 (48.7%) reported data on older adults. Trends in demographic reporting over time are shown in **Figure 1A**. White participants remained the majority but declined from 77.2% in 2018 to 64.3% in 2024. Female representation varied between 30.8% (2019) and 43.6% (2024). Hispanic participants increased steadily from 4.7% in 2018 to 13.1% in 2023, while Black participants rose from 2.7% (2019) to 3.6% (2023). Asian or Pacific Islander representation peaked at 27.9% in 2024. Excluding pediatric trials, older adults accounted for 30.7% to 52.6% of participants, with the highest proportion observed in 2023 (**Figure 1B**). The statistical trend analysis for these changes is presented below in the meta-regression section.

### Demographic disparity between trial and US population

**Figure 2:**
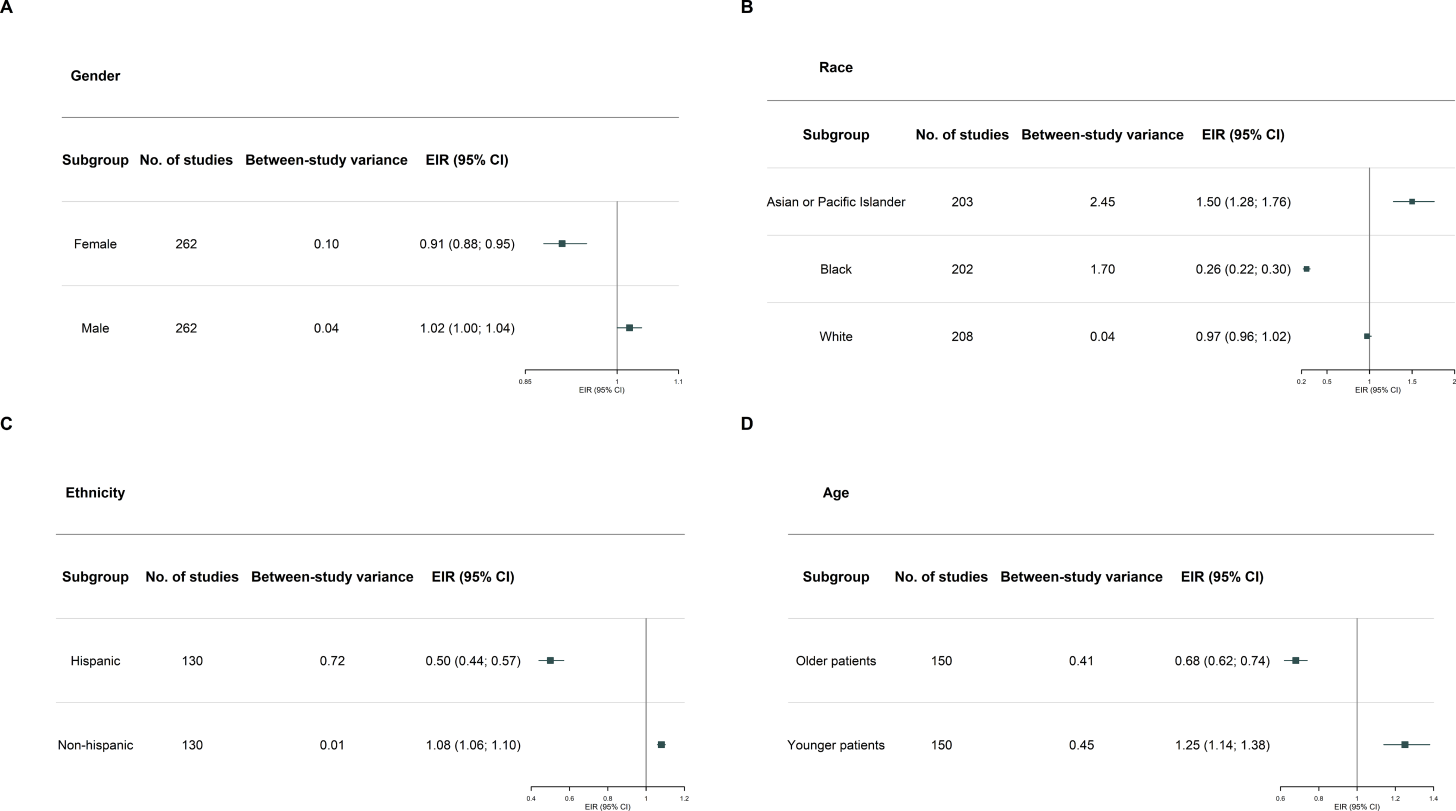
Forest plots showing the meta-analysis of enrollment incidence ratios (EIRs) by **(A)** gender, **(B)** race, **(C)** ethnicity, and **(D)** age. An EIR greater than 1 indicates overrepresentation, whereas an EIR less than 1 indicates underrepresentation compared with population-based estimates.

Regarding gender representation, analysis of 98 trials showed that female participants were significantly underrepresented (EIR, 0.83; 95% CI, 0.78–0.88), while male participants were overrepresented (EIR, 1.12; 95% CI, 1.07–1.16) (**Figure 2A**).

As shown in **Figure 2B**, White participants were proportionally represented (EIR, 0.97; 95% CI, 0.96–1.02), while Black participants (EIR, 0.26; 95% CI, 0.22–0.30) were significantly underrepresented. In contrast, Asian or Pacific Islander participants were overrepresented (EIR, 1.50; 95% CI, 1.28–1.76).

Ethnicity data from 130 trials showed substantial underrepresentation of Hispanic individuals (EIR, 0.50; 95% CI, 0.44–0.57), whereas non-Hispanic participants were overrepresented (EIR, 1.08; 95% CI, 1.06–1.10) (**Figure 2C**).

Similarly, among 110 trials, older adults were underrepresented (EIR, 0.86; 95% CI, 0.83–0.89), suggesting a disproportionate enrollment of younger individuals (EIR, 1.25; 95% CI, 1.14–1.38) (**Figure 2D**).

### Subgroup and sensitivity analyses

**Figure 3:**
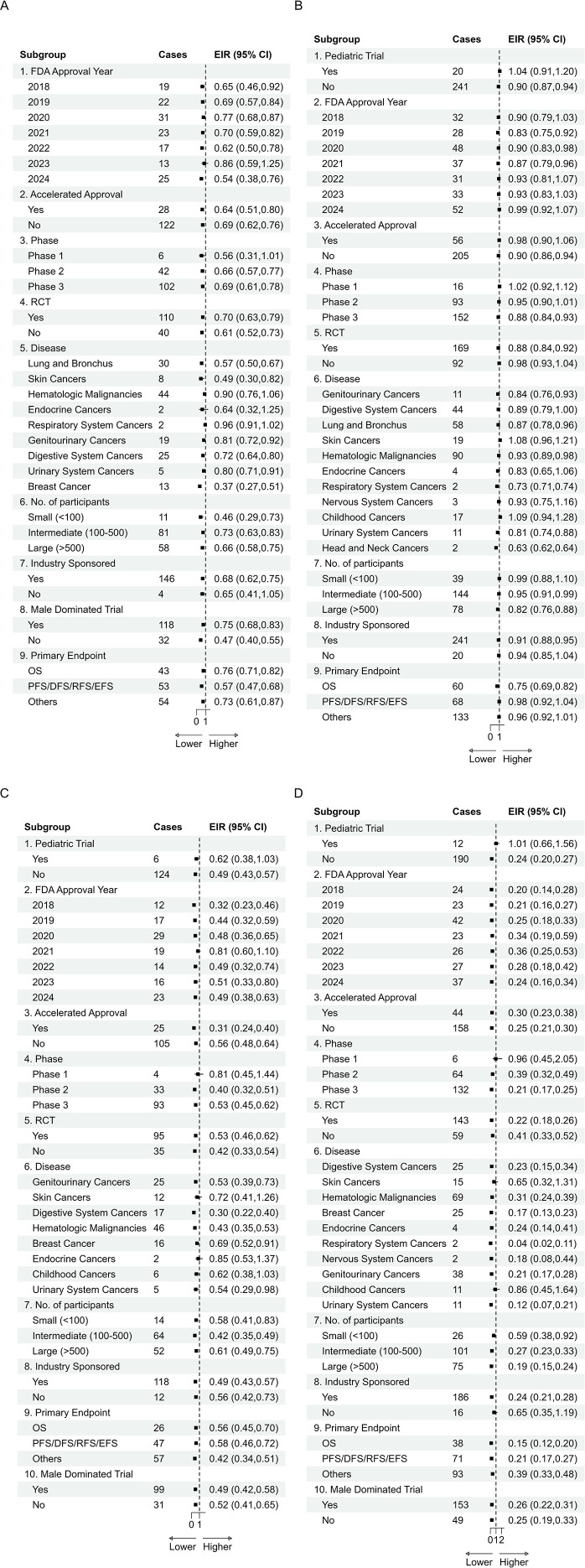
Subgroup analysis results. **(A)** Subgroup of older patients; **(B)** Subgroup of female patients; **(C)** Subgroup of Hispanic patients; **(D)** Subgroup of Black patients. To facilitate interpretation of the dense subgroup forest plots in **Figure 3**, readers may first compare each panel’s pooled estimates to the EIR = 1 reference line and then assess whether the direction of under- or over-enrollment is consistent across major trial features (accelerated approval, phase, randomization, and endpoint). Overall, estimates were directionally consistent across most subgroups, supporting the robustness of the primary findings; however, results for strata with few contributing trials are less precise and should be interpreted cautiously.

As shown in **Figure 3**, in pediatric trials, representation of female participants (EIR, 1.04; 95% CI, 0.91–1.20), Black participants (EIR, 1.01; 95% CI, 0.66–1.56), Asian participants (EIR, 1.14; 95% CI, 0.63–2.07), and non-Hispanic individuals (EIR, 1.12; 95% CI, 1.02–1.22) was approximately proportional. However, Hispanic participants (EIR, 0.62; 95% CI, 0.38–1.03) and White participants (EIR, 0.91; 95% CI, 0.80–1.05) remained underrepresented. In accelerated approval trials, female and male participants were proportionally represented (EIRs, 0.98 and 0.97, respectively), while Black (EIR, 0.30; 95% CI, 0.23–0.38), Hispanic (EIR, 0.31; 95% CI, 0.24–0.40), and older adults (EIR, 0.64; 95% CI, 0.51–0.80) were markedly underrepresented.

For other subgroups, female participants were underrepresented in most subgroups. However, this trend was not seen in phase 1 trials (EIR, 1.02; 95% CI, 0.92–1.12), skin cancer trials (EIR, 1.08; 95% CI, 0.96–1.21), childhood cancer trials (EIR, 1.09; 95% CI, 0.94–1.28), where representation was approximately proportional. Black participants were persistently underrepresented across all subgroups, with the lowest EIRs observed in trials for respiratory system cancers (EIR, 0.04; 95% CI, 0.02–0.11). Underrepresentation of Hispanic participants and older adults observed in the primary analysis was broadly consistent across subgroups. Detailed results are provided in **Figure 3**. Subgroup-specific results for male participants, younger adults, non-hispanic participants, White individuals, and Asian or Pacific Islander individuals are presented in **eTables 3**-**7**.

Sensitivity analyses further confirmed the robustness of primary findings. When including trials across all indications, Black participants remained markedly underrepresented (EIR, 0.22; 95% CI, 0.19–0.25), White participants were proportionally represented (EIR, 0.91; 95% CI, 0.88–0.94), and Asian or Pacific Islander participants were significantly overrepresented (EIR, 1.91; 95% CI, 1.65–2.20). Among ethnic subgroups, Hispanic participants were underrepresented (EIR, 0.50; 95% CI, 0.44–0.57), whereas non-Hispanic participants were slightly overrepresented (EIR, 1.07; 95% CI, 1.06–1.09). Furthermore, using Black-to-White enrollment ratios, Black representation remained consistently low (EIR, 0.37; 95% CI, 0.31–0.44) (**eFigure 1**).

### Meta-regression analyses results

**Figure 4:**
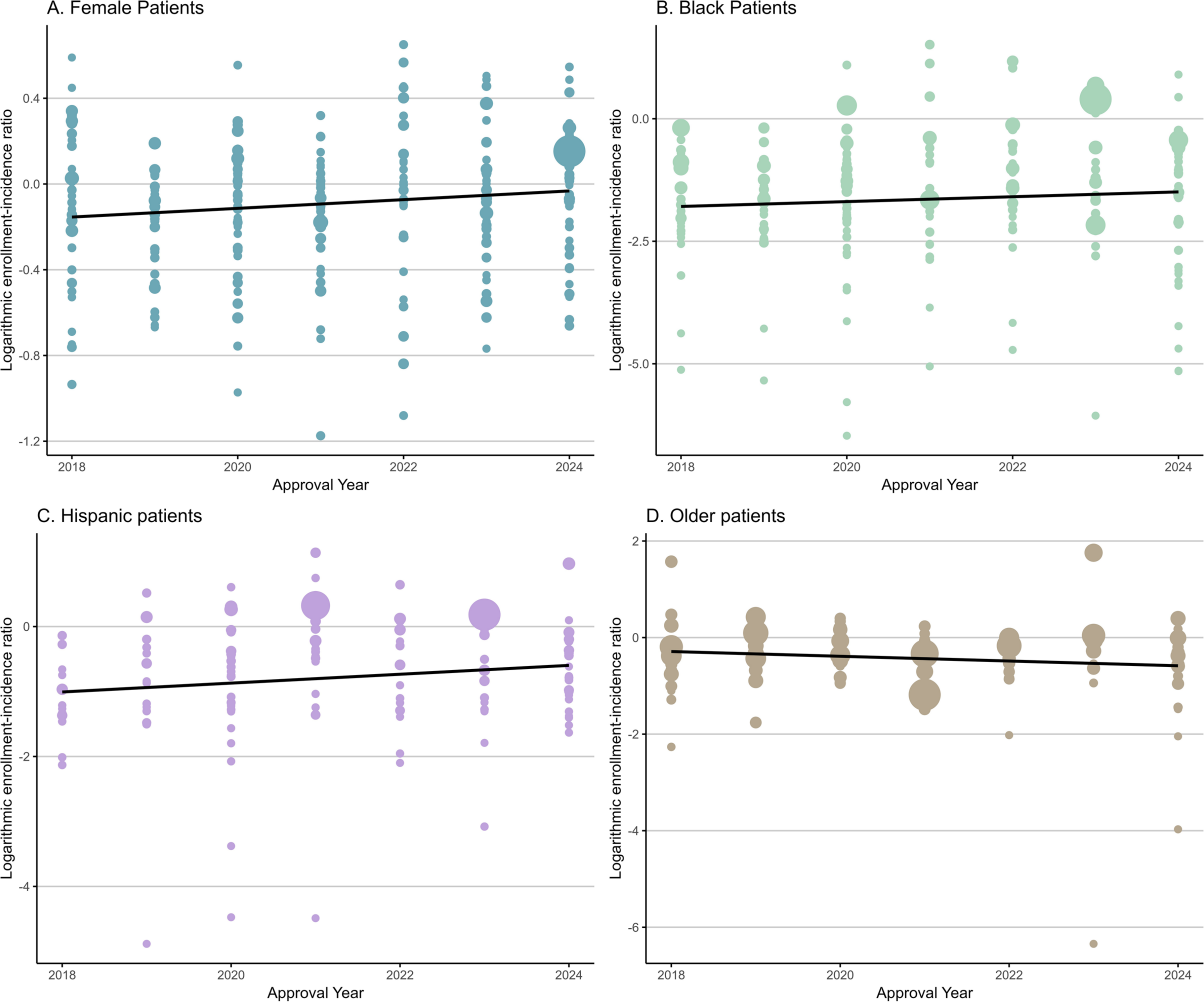
Trends in enrollment disparities in cancer clinical trials for **(A)** female patients, **(B)** Black patients, **(C)** Hispanic patients, and **(D)** older patients. To facilitate interpretation of **Figure 4**, readers may first focus on the fitted trend line in each panel to assess the direction and magnitude of temporal change in enrollment disparities, and then use the vertical spread of points to gauge between-trial heterogeneity within each approval year. Point size reflects trial size, so larger circles correspond to estimates that are more influential; overall, the fitted lines suggest broadly stable disparities over time with only modest year-to-year changes, and isolated extreme values should be interpreted cautiously.

We examined four patient groups that have been historically underrepresented in clinical trials: female, older, Hispanic, and Black participants. Univariable meta-regression analysis revealed a significant increase in the enrollment of female patients over time (β=0.02, p=0.03), suggesting meaningful progress in addressing gender disparities. While participation among Black (β=0.05, p=0.21) and Hispanic (β=0.04, p=0.21) patients also showed upward trends, these changes did not reach statistical significance. For older adults, enrollment slightly decreased (β=–0.03, p =0.29) (**Figure 4A–D**).

Multivariable meta-regression (**eTable 8**) demonstrated that representation of older adults was significantly higher in male-dominated trials (β=0.48, p<0.001). Hispanic participants were significantly underrepresented in trials granted accelerated approval (β=-0.55, p=0.005). Female underrepresentation was significantly greater in trials employing OS as the primary endpoint (β=0.11, p=0.003). Black participants were significantly less represented in phase 3 trials compared to phase 1–2 (β=-0.68, p=0.003) and in industry-sponsored trials (β=-0.64, p=0.01), but significantly more represented in trials utilizing non-OS endpoints (β=0.28, p=0.03) and in pediatric trials (β=0.90, p=0.002). No evidence of multicollinearity was observed, more information is in **eTable 9**.

Study-level EIRs did not differ significantly between pandemic and non-pandemic periods for most subgroups. EIRs were lower during the pandemic for adults aged over 65 years (median, 0.64 vs 0.76; P=0.05) and for White participants (median, 1.00 vs 1.05; P=0.02); no differences were observed for other subgroups. More information is in **eTable 10**.

## Discussion

To our knowledge, this is the first study to systematically evaluate demographic disparities in pivotal oncology trials supporting FDA approvals in the post-2018 policy era. Prior analyses have largely focused on earlier periods or specific cancer types, without addressing the evolving structural and regulatory landscape of modern oncology trials. Despite growing regulatory focus on diversity (8, 9), women, older adults, Black, and Hispanic patients remain consistently underrepresented. These disparities highlight a continued disconnect between trial populations and the demographics of the US cancer burden, raising concerns about the generalizability of findings and the equitable delivery of new cancer therapies. As precision medicine advances, representative enrollment is essential for both scientific validity and health equity (20).

While some trials included international enrollment, our use of SEER incidence estimates is justified by the focus on FDA-approved therapies intended for the US population, and sensitivity analyses were conducted to account for regionally prevalent diseases (6).

Female participants remained underrepresented in pivotal oncology trials. This disparity, consistently reported in prior studies (21), likely reflects multifactorial barriers, including restrictive eligibility criteria, recruitment practices, and differential access to care. In our analysis, use of OS as the primary endpoint was associated with lower female enrollment. This pattern may reflect differences in trial design, follow-up burden, or eligibility requirements (eg, performance status thresholds), although our study cannot establish causality. OS-driven registration trials often require longer follow-up and more frequent assessments, which may increase time and travel burdens and differentially affect participation. In addition, OS-focused designs may adopt more restrictive eligibility criteria to minimize competing risks and loss to follow-up, which could influence who is able to enroll. These explanations are speculative and our analyses identify associations rather than causal effects. Given known sex-based differences in cancer biology, pharmacokinetics, and toxicity profiles (22–24), inadequate female inclusion limits the generalizability and safety assessment of oncology therapies.

Our analysis shows clear underrepresentation of Black and Hispanic patients in pivotal oncology trials, with enrollment ratios falling well below their cancer burden. These disparities reflect longstanding structural barriers—such as access to academic centers, socioeconomic challenges, and limited community engagement in recruitment. Given known differences in tumor biology and drug response across populations, inadequate representation hinders accurate assessment of treatment efficacy and safety in underserved groups (4).

Older adults, who comprise the majority of cancer patients, remained underrepresented in pivotal oncology trials. Across studies, the median age of trial participants consistently fell below the population median at diagnosis, reflecting a persistent age bias (7). This underrepresentation may stem from stringent eligibility criteria, concerns over comorbidities, polypharmacy, or physician reluctance to enroll older individuals (25). Beyond these factors, as summarized by Sedrak et al (7), narrow eligibility, trial complexity, and limited geriatric-focused studies restrict access, while providers’ toxicity concerns and tumor biology favoring younger patients further exacerbate disparities. Patient preferences, logistical burdens, and caregiver strain also contribute. The COVID-19 pandemic likely intensified these barriers by limiting mobility and digital access. Together, these factors underscore the need for age-inclusive and pragmatic trial designs and greater use of real-world data to inform evidence generation in geriatric oncology (7). Exclusion of older adults undermines the generalizability of trial findings, particularly as this group often experiences distinct pharmacodynamics, treatment-related toxicity, and benefit-risk trade-offs (26). As the oncology landscape increasingly shifts toward individualized care, capturing the heterogeneity of aging populations in clinical research is essential to inform safe and effective treatment strategies.

Randomization has been reported as a barrier to trial participation in prior studies. In this study, non-randomized designs mainly occurred in early-phase or accelerated approval settings. The increasing use of single-arm studies reflects the FDA’s support for expedited access to promising therapies, particularly for rare or biomarker-defined cancers. However, such designs are often conducted at specialized centers and may involve narrower eligibility criteria, which may be associated with less diverse enrollment. Consistent with this, we observed lower representation of several underrepresented groups in certain trial settings, although these associations do not imply causality. Ensuring that future expedited and early-phase trials incorporate inclusive enrollment strategies will be essential to balance efficiency with equity in oncology drug development.

Encouragingly, our meta-regression analysis suggests a modest improvement in demographic representation over time, with trial populations becoming incrementally more reflective of the broader cancer population. However, disparities persist, particularly among racial and ethnic minorities. Black and Hispanic patients remained markedly underrepresented, with no statistically significant gains observed in their enrollment. Our findings also highlight the role of trial-level factors in shaping participant diversity. Industry-sponsored trials were associated with lower representation of Black participants, while two-arm designs were linked to reduced Hispanic enrollment. This association may reflect sponsor-driven operational considerations, including selection of sites within established research networks and an emphasis on rapid recruitment timelines, which may concentrate enrollment in large academic centers. Such site distributions can be less connected to community or safety-net settings that serve a higher proportion of underrepresented patients. We did not have site-level data to directly test these mechanisms. These patterns are consistent with prior work demonstrating that trial sponsorship, complexity, and endpoint selection can influence accessibility and inclusivity (27, 28). Addressing these structural factors through intentional trial design and regulatory oversight will be critical to advancing equity in cancer research. Importantly, the meta-regression analyses identify trial-level associations and should not be interpreted as evidence of causal effects.

The persistent underrepresentation of diverse populations in registration trials supporting FDA approval has significant implications for health equity. Without inclusive enrollment, clinical trial results may fail to capture differences in treatment efficacy and toxicity across demographic subgroups, potentially reinforcing disparities in access to and benefit from newly approved therapies (29). Addressing these gaps requires a multifaceted approach: inclusive trial designs, community engagement to build trust among underrepresented populations, and strict adherence to federal diversity mandates (30, 31). As regulatory agencies increasingly emphasize demographic transparency and equity, ensuring representative participation is both a scientific and ethical imperative.

### Impact of the COVID-19 pandemic on longitudinal interpretation

The study period (2018–2024) encompassed the COVID-19 pandemic, which substantially disrupted the conduct of oncology clinical trials worldwide. During COVID-19 pandemic, many programs experienced temporary trial suspensions, patient-recruitment challenges, and rapid shifts toward decentralized or virtual trial designs. These operational changes may have influenced participant demographics and trial conduct. Consequently, part of the observed year-to-year variation in subgroup representation might reflect pandemic-related factors rather than underlying systematic changes in enrollment practices. Findings from the American Society of Clinical Oncology survey similarly reported that numerous US cancer research programs temporarily halted or prioritized trial enrollment, adopted remote patient monitoring, and restructured staff operations during the early stages of the pandemic. Such disruptions could have affected access to clinical trials for certain racial and ethnic minority groups, older adults, and other underserved populations (32). Although our analysis evaluated temporal trends across 2018-2024, the pandemic years may have introduced short-term shocks that complicate longitudinal interpretation; therefore, meta-regression-based trends should be interpreted cautiously.

### Alignment with US regulatory initiatives

Our findings have direct relevance to recent US regulatory initiatives to improve trial diversity. The 2022 FDORA authorizes the FDA to require DAP for certain pivotal trials, including enrollment goals and strategies by sex, race, ethnicity, and age. Nevertheless, we observed only modest improvement in female representation and no statistically significant gains for Black or Hispanic participants, with older-adult representation showing a slight decline. Several factors may explain this apparent lack of improvement: FDORA was enacted late in the study period, and many pivotal trials supporting approvals in 2022–2024 likely initiated enrollment before FDORA implementation; DAP submission does not guarantee attainment of targets in the presence of persistent structural barriers (eg, restrictive eligibility criteria, site selection, and access constraints); and multinational recruitment may further limit alignment between trial populations and the US cancer population. These results underscore the need for timely implementation, transparent reporting, and accountability mechanisms to translate DAP requirements into measurable improvements in representativeness.

### Future directions

Improving equity in pivotal oncology trials will require coordinated efforts across trial design, regulatory oversight, and community engagement. Future research should prioritize the implementation and evaluation of strategies to enhance demographic diversity, including the systematic broadening of eligibility criteria, decentralization of trial infrastructure, and targeted outreach to historically underrepresented populations (33). Establishing trial sites in community-based and safety-net settings may improve access for minority, low-income, and older patients (30). In parallel, consistent and detailed reporting of participant characteristics—by race, ethnicity, sex, and age—is essential to monitor progress and identify persistent gaps. Regulatory authorities and sponsors must move beyond guidance toward enforceable accountability frameworks, including the use of Diversity Action Plans and performance-linked incentives to promote inclusive enrollment (34). These measures are critical to ensuring that clinical trial evidence reflects the populations most affected by cancer and supports equitable translation into clinical practice.

### Limitations

This study has several limitations. First, reliance on publicly available sources, including ClinicalTrials.gov and SEER, may have introduced reporting bias due to missing or incomplete data, particularly from unpublished trials or undocumented cancer cases. Second, racial groups with low population numbers, such as American Indian and Alaska Native individuals, were not included in subgroup analyses, limiting the generalizability of findings to these populations. Third, the analysis focused on trials supporting FDA approval of cancer treatments, which may not reflect representational patterns in global oncology research. Fourth, the inclusion of a wide range of cancer types, therapeutic classes, and trial designs across multiple years introduces clinical and methodological heterogeneity. Importantly, different malignancies inherently differ in demographic composition (eg, sex distribution and age at diagnosis), and racial/ethnic distributions may also vary by cancer type. As a result, pooled EIRs should be interpreted as overall averages across tumor types and may attenuate or mask disparities that are specific to certain cancers or settings, despite the use of stratified and sensitivity analyses. Fifth, many pivotal trials supporting FDA approvals were multinational, especially industry-sponsored studies. Because our aim is to assess representativeness for US patients, global recruitment is an inherent limitation of the registration evidence base and may partly explain observed gaps when trials are benchmarked to SEER. Sixth, Incomplete demographic reporting is another limitation. Because EIR estimates were based on trials reporting the relevant variable, our results may be influenced by selective reporting and may not fully reflect all pivotal trials supporting FDA approvals. Finally, some pivotal trials included international study sites, including centers in Asia, which may have modestly increased the proportion of Asian participants; this geographic factor may partly contribute to the observed overrepresentation of Asian or Pacific Islander individuals.

## Conclusion

In this study of pivotal clinical trials supporting FDA oncology drug approvals from 2018 to 2024, we observed persistent underrepresentation of women, older adults, Black, and Hispanic patients. Although recent federal policies have aimed to improve trial diversity, disparities remain widespread, with only slight improvements over time. Addressing these gaps may require changes in trial eligibility criteria, recruitment practices, and site selection to ensure study populations more accurately reflect the US cancer population.

## Data Availability

The original contributions presented in the study are included in the article/**Supplementary Material**. Further inquiries can be directed to the corresponding author.
