# Supplementary material for: Equity and representation in oncology clinical evidence for FDA-approved treatments and the impact of COVID-19
Source: Front Oncol. 2026 May 1;16:1666497. doi: 10.3389/fonc.2026.1666497 (PMC13175864; doi:10.3389/fonc.2026.1666497)
Supplement: Supplementary file 1 [file DataSheet1.docx]

**[Supplement](#_Toc186664319)**

[eMethod Detailed data extraction and analysis, selection of variables for meta-regression, and pandemic-related analysis 2](#_Toc9126)

[eTable 1 Summary of FDA-Approved Oncology Therapies and Supporting Trials, 2018–2024 4](#_Toc644)

[eTable 2 Subgroup Categories and Definitions 14](#_Toc12571)

[eFigure 1 Sensitivity Meta-Analysis of Enrollment Incidence Ratios (EIRs) 15](#_Toc9711)

[eTable 3 Subgroup Analysis Results for Male Participant 16](#_Toc24349)

[eTable 4 Subgroup Analysis Results for White Participant 18](#_Toc24647)

[eTable 5 Subgroup Analysis Results for Asian or Pacific Islander individuals 21](#_Toc15260)

[eTable 6 Subgroup Analysis Results for non-Hispanic individuals 24](#_Toc7763)

[eTable 7 Subgroup Analysis Results for younger adults 26](#_Toc9333)

[eTable 8 Results of multivariable meta-regression 28](#_Toc8684)

[eTable 9 Multicollinearity Analysis Results 29](#_Toc27109)

[eTable 10 Study-level EIRs: Pandemic (enrollment midpoint 2020-2022) vs non-pandemic 29](#_Toc16655)

[Figure S1. Study selection flow diagram 30](#_Toc7618)

# eMethod Detailed data extraction and analysis, selection of variables for meta-regression, and pandemic-related analysis

1. Eligibility criteria

Two reviewers (Z.M. and J.Y.) independently extracted data from eligible trial full-text publications. If unavailable, supplementary materials or ClinicalTrials.gov were used.

For each trial, the following characteristics were collected: trial identification, median recruitment year, publication year, trial phase, disease class, clinical setting, sample size, number of arms, treatment regimen class, control regimen, funding source, and primary endpoints.

Demographic data were extracted per the FDA position statement, including distribution of gender (female, male), race (African American/Black, Asian, American Indian/Alaskan Native, Native Hawaiian/Other Pacific Islander, White), ethnicity (Hispanic/Latino, non-Hispanic/non-Latino), and age (≥65 vs <65 years).

1. Quantification of imprecision in data analysis

In certain clinical trials where the number of participants from specific populations is zero (e.g., the number of Black participants in small-sample studies), we assume a value of 0.1 for the calculation of the Enrollment Incidence Ratio (EIR). This approach, commonly referred to as a continuity correction, is widely utilized in statistical analyses to avoid computational issues associated with zero counts and to stabilize ratio estimates, particularly in small-sample studies.^[1, 2]^ We excluded trials with zero participants from these specific populations to assess the robustness of our findings in sensitivity analyses.

To quantify the imprecision of EIR across gender, racial, and ethnic categories, according to Irbaz et al,^[3]^ and used the following formula to calculate the standard error (SE) of logEIRs:

$$SE=\sqrt{(\frac{1}{a_{1}}-\frac{1}{n_{1}})+(\frac{1}{a_{2}}-\frac{1}{n_{2}}})$$

$a_{1}$ = the number of participants in each relevant category of a subgroup

$a_{2}$ = the total number of enrolled participants in the trial

$n_{1}$ = the number of US patients diagnosed with specific cancer in each relevant category of a subgroup

$n_{2}$ = the total number US patients diagnosed with specific cancer.

Confidence intervals for EIRs

For each trial, we computed the enrollment incidence ratio (EIR) and analyzed effects on the logarithmic scale. Let logEIR = ln(EIR) with standard error SE(logEIR) calculated as described above. The 95% confidence interval (CI) on the log scale was calculated as:

logEIR ± 1.96 × SE(logEIR).

We then back-transformed the CI to the original scale as:

(exp[logEIR − 1.96×SE], exp[logEIR + 1.96×SE]).

1. Meta-analysis and Meta-regression

To construct the final multivariable model, we first reviewed the existing literature to identify factors previously reported as predictors of racial/ethnic or age disparities in clinical trials. Based on this, we selected the following trial characteristics for inclusion in the multivariable meta-regression: number of trial phase,^[4]^ tumor type (categorized into five groups based on 5-year survival rates from SEER 21, using <20%, 20–40%, 40–60%, 60–80%, and ≥80% as cutoffs),^[4]^ trial size (number of patients enrolled),^[3]^ funding source,^[5]^ and primary endpoint.^[6]^ Additionally, the number of trial arms was included as a variable to account for its potential role as a confounder in the association between trial phase and enrollment disparity, ensuring proper adjustment for its potential influence. Other trial-level covariates included in the final model were randomization status (randomized controlled trial vs non-randomized),^[7]^ male-dominated enrollment (defined as ≥70% male participants),^[4]^ pediatric trial status,^[8]^ accelerated approval designation,^[9]^ and FDA approval year.^[10]^

Handling heterogeneity

We synthesized logEIRs using random-effects models to account for between-trial heterogeneity in underlying representativeness across cancer types, trial designs, and enrollment settings. Between-study variance was summarized using τ², and the proportion of variability attributable to between-study heterogeneity was quantified using I². To explore potential sources of heterogeneity, we conducted prespecified subgroup analyses and meta-regression using trial-level covariates. τ² was estimated using restricted maximum likelihood.

1. Handling of missing demographic reporting

We did not impute missing demographic fields. For each demographic subgroup outcome (eg, sex, race, ethnicity, age), analyses were restricted to trials that reported sufficient information to compute the corresponding EIR. Trials with missing reporting for a given demographic field were excluded from that specific analysis but remained eligible for other analyses with available demographic data.

1. Demographic reporting rates over time.

Because demographic reporting (eg, ethnicity) was incomplete and varied across trials and calendar time, estimates involving a given demographic variable—including time-trend meta-regression—were performed using the subset of trials that reported that variable; as a result, the number of included trials may differ across outcomes and years.

1. Pandemic period definition and analysis.

To examine whether demographic representation differed during the COVID-19 pandemic, we compared study-level enrollment inequality ratios (EIRs) between trials enrolling during the pandemic period and those enrolling outside the pandemic period. We defined the pandemic period a priori as calendar years 2020–2022, reflecting the interval in which oncology trial conduct and patient participation were plausibly most affected by COVID-19–related disruptions (eg, site access restrictions, staffing constraints, reduced in-person visits, and differential barriers to participation), and capturing both the initial acute phase (2020) and subsequent waves and recovery phases (2021–2022).

For each subgroup, we summarized study-level EIRs using the median and interquartile range (IQR) because EIR distributions were skewed. We compared pandemic vs non-pandemic study-level EIRs using a two-sided Wilcoxon rank-sum test (Mann–Whitney U) with P<.05 considered statistically significant. We additionally estimated Cliff’s delta with 95% CIs as a nonparametric effect size; negative values indicate lower EIRs among trials whose enrollment occurred during the pandemic period.

Assignment of trials to calendar periods. Each trial was assigned a calendar period using the median year of patient enrollment (enrollment midpoint year) derived from the trial’s enrollment start and end dates. Trials with an enrollment midpoint in 2020–2022 were classified as “pandemic,” and those with enrollment midpoints outside this window were classified as “non-pandemic.”

# eTable 1 Summary of FDA-Approved Oncology Therapies and Supporting Trials, 2018–2024

| Number | FDA Approval Year | Approved Therapy | Supporting Trial (NCT No.) |
| --- | --- | --- | --- |
| 1 | 2024 | nivolumab and hyaluronidase-nvhy | CHECKMATE-67T (NCT04810078) |
| 2 | 2024 | encorafenib with cetuximab and mFOLFOX6 | BREAKWATER (NCT04607421) |
| 3 | 2024 | ensartinib | eXALT3 (NCT02767804) |
| 4 | 2024 | cosibelimab-ipdl | Study CK-301-101 (NCT03212404) |
| 5 | 2024 | durvalumab | ADRIATIC (NCT03703297) |
| 6 | 2024 | zenocutuzumab-zbco | eNRGy study (NCT02912949) |
| 7 | 2024 | zanidatamab-hrii | HERIZON-BTC-01 (NCT04466891) |
| 8 | 2024 | revumenib | SNDX-5613-0700, NCT04065399 |
| 9 | 2024 | obecabtagene autoleucel | FELIX (NCT04404660) |
| 10 | 2024 | asciminib | ASC4FIRST (NCT04971226) |
| 11 | 2024 | zolbetuximab-clzb | SPOTLIGHT (NCT03504397) |
| 12 | 2024 | zolbetuximab-clzb | GLOW (NCT03653507) |
| 13 | 2024 | inavolisib with palbociclib | INAVO120 (NCT04191499) |
| 14 | 2024 | nivolumab | CHECKMATE-77T (NCT04025879) |
| 15 | 2024 | selpercatinib | LIBRETTO-531 (NCT04211337) |
| 16 | 2024 | osimertinib | LAURA (NCT03521154) |
| 17 | 2024 | isatuximab-irfc with bortezomib, lenalidomide, and dexamethasone | IMROZ (NCT03319667) |
| 18 | 2024 | amivantamab-vmjw with carboplatin and pemetrexed | MARIPOSA-2 (NCT04988295) |
| 19 | 2024 | pembrolizumab with pemetrexed and platinum chemotherapy | KEYNOTE-483 (NCT02784171) |
| 20 | 2024 | ribociclib with an aromatase inhibitor | NATALEE (NCT03701334) |
| 21 | 2024 | atezolizumab and hyaluronidase-tqjs | IMscin001 (NCT03735121) |
| 22 | 2024 | lazertinib in combination with amivantamab-vmjw | MARIPOSA (NCT04487080) |
| 23 | 2024 | durvalumab with platinum-containing chemotherapy | AEGEAN (NCT03800134) |
| 24 | 2024 | vorasidenib | INDIGO (NCT04164901) |
| 25 | 2024 | afamitresgene autoleucel | SPEARHEAD-1, Cohort 1 |
| 26 | 2024 | dostarlimab-gxly with carboplatin and paclitaxel, followed by single-agent dostarlimab-gxly | RUBY (NCT03981796) |
| 27 | 2024 | daratumumab and hyaluronidase-fihj in combination with bortezomib, lenalidomide, and dexamethasone | PERSEUS (NCT03710603) |
| 28 | 2024 | epcoritamab-bysp | EPCORE NHL-1 (Study GCT3013-01; NCT03625037) |
| 29 | 2024 | adagrasib with cetuximab | KRYSTAL-1,NCT03785249 |
| 30 | 2024 | pembrolizumab with chemotherapy | KEYNOTE-868/NRG-GY018 (NCT03914612) |
| 31 | 2024 | blinatumomab | Study E1910 (NCT02003222) |
| 32 | 2024 | durvalumab with chemotherapy | DUO-E (NCT04269200) |
| 33 | 2024 | repotrectinib | TRIDENT-1 (NCT03093116) |
| 34 | 2024 | selpercatinib | LIBRETTO-001 (NCT03157128) |
| 35 | 2024 | imetelstat | IMerge (NCT02598661) |
| 36 | 2024 | lisocabtagene maraleucel | TRANSCEND-MCL (NCT02631044) |
| 37 | 2024 | tarlatamab-dlle | DeLLphi-301 [NCT05060016] |
| 38 | 2024 | lisocabtagene maraleucel | TRANSCEND-FL (NCT04245839) |
| 39 | 2024 | tisotumab vedotin-tftv | innovaTV 301 (NCT04697628) |
| 40 | 2024 | tovorafenib | FIREFLY-1 (NCT04775485) |
| 41 | 2024 | lutetium Lu 177 dotatate | NETTER-1 (NCT01578239) |
| 42 | 2024 | lutetium Lu 177 dotatate | NETTER-P (NCT04711135) |
| 43 | 2024 | nogapendekin alfa inbakicept-pmln | QUILT-3.032 (NCT0302285) |
| 44 | 2024 | alectinib | ALINA, NCT03456076 |
| 45 | 2024 | fam-trastuzumab deruxtecan-nxki | DESTINY-Lung01 (NCT03505710) |
| 46 | 2024 | fam-trastuzumab deruxtecan-nxki | DESTINY-CRC02 (NCT04744831) |
| 47 | 2024 | mirvetuximab soravtansine-gynx | Study 0416 (MIRASOL, NCT04209855) |
| 48 | 2024 | ponatinib with chemotherapy | PhALLCON (NCT03589326) |
| 49 | 2024 | zanubrutinib with obinutuzumab | Study BGB-3111-212 (ROSEWOOD; NCT03332017) |
| 50 | 2024 | nivolumab in combination with cisplatin and gemcitabine | CHECKMATE-901 (NCT03036098) |
| 51 | 2024 | inotuzumab ozogamicin | Study WI203581 (ITCC-059; NCT02981628) |
| 52 | 2024 | amivantamab-vmjw | PAPILLON (NCT04538664) |
| 53 | 2024 | osimertinib with chemotherapy | FLAURA 2 (NCT04035486) |
| 54 | 2024 | lifileucel | C-144-01 trial (NCT02360579) |
| 55 | 2024 | tepotinib | VISION trial (NCT02864992) |
| 56 | 2024 | irinotecan liposome | NAPOLI 3 (NCT04083235) |
| 57 | 2024 | erdafitinib | Study BLC3001 Cohort 1 |
| 58 | 2024 | pembrolizumab with chemoradiotherapy | KEYNOTE-A18 (NCT04221945) |
| 59 | 2023 | enfortumab vedotin-ejfv with pembrolizumab | EV-302/KN-A39 (NCT04223856) |
| 60 | 2023 | belzutifan | LITESPARK-005 (NCT04195750) |
| 61 | 2023 | eflornithine | Study 3b (NCT02395666) |
| 62 | 2023 | eflornithine | Study ANBL0032 (clinical trial-derived external control arm) |
| 63 | 2023 | pirtobrutinib | BRUIN (NCT03740529) |
| 64 | 2023 | enzalutamide | EMBARK (NCT02319837) |
| 65 | 2023 | capivasertib with fulvestrant | CAPItello-291 (NCT04305496) |
| 66 | 2023 | embrolizumab with chemotherapy | KEYNOTE-859 (NCT03675737) |
| 67 | 2023 | repotrectinib | TRIDENT-1, NCT03093116 |
| 68 | 2023 | fruquintinib | FRESCO-2 (NCT04322539) |
| 69 | 2023 | pembrolizumab | KEYNOTE-811 (NCT03615326) |
| 70 | 2023 | pembrolizumab with chemotherapy | KEYNOTE-966 (NCT04003636) |
| 71 | 2023 | toripalimab-tpzi | JUPITER-02 (NCT03581786) |
| 72 | 2023 | Ivosidenib | AG120-C-001 (NCT02074839) |
| 73 | 2023 | pembrolizumab | KEYNOTE-671 (NCT03425643) |
| 74 | 2023 | nivolumab | CHECKMATE-76K (NCT04099251) |
| 75 | 2023 | encorafenib with binimetinib | PHAROS (NCT03915951) |
| 76 | 2023 | bosutinib | BCHILD trial (NCT04258943) |
| 77 | 2023 | melphalan | FOCUS study (NCT02678572) |
| 78 | 2023 | elranatamab-bcmm | MagnetisMM-3 (NCT04649359) |
| 79 | 2023 | niraparib and abiraterone acetate plus prednisone | Cohort 1 of MAGNITUDE (NCT03748641) |
| 80 | 2023 | talquetamab-tgvs | MMY1001 (MonumenTAL-1) (NCT03399799) |
| 81 | 2023 | pralsetinib | ARROW trial (NCT03037385) |
| 82 | 2023 | trifluridine and tipiracil | SUNLIGHT (NCT04737187) |
| 83 | 2023 | dostarlimab-gxly with chemotherapy | RUBY (NCT03981796) |
| 84 | 2023 | quizartinib | QuANTUM-First (NCT02668653) |
| 85 | 2023 | talazoparib with enzalutamide | TALAPRO-2 (NCT03395197) |
| 86 | 2023 | glofitamab-gxbm | NP30179 (NCT03075696) |
| 87 | 2023 | olaparib with abiraterone and prednisone (or prednisolone) | PROpel trial (NCT03732820) |
| 88 | 2023 | epcoritamab-bysp | EPCORE NHL-1 (NCT03625037) |
| 89 | 2023 | polatuzumab vedotin-piiq | POLARIX (NCT03274492) |
| 90 | 2023 | omidubicel | Study P0501 (NCT02730299) |
| 91 | 2023 | enfortumab vedotin-ejfv with pembrolizumab | EV-103/KEYNOTE-869 (NCT03288545) |
| 92 | 2023 | retifanlimab-dlwr | PODIUM-201 (NCT03599713) |
| 93 | 2023 | dabrafenib with trametinib | Study CDRB436G2201 (NCT02684058) |
| 94 | 2023 | abemaciclib with endocrine therapy | monarchE (NCT03155997) |
| 95 | 2023 | dostarlimab-gxly | GARNET (NCT02715284) |
| 96 | 2023 | sacituzumab govitecan-hziy | TROPiCS-02 (NCT03901339) |
| 97 | 2023 | elacestrant | EMERALD (NCT03778931) |
| 98 | 2023 | pirtobrutinib | BRUIN (NCT03740529) |
| 99 | 2023 | pembrolizumab | KEYNOTE-091 (NCT02504372) |
| 100 | 2023 | zanubrutinib | SEQUOIA (NCT03336333) |
| 101 | 2023 | zanubrutinib | ALPINE (NCT03734016) |
| 102 | 2023 | tucatinib with trastuzumab | MOUNTAINEER (NCT03043313) |
| 103 | 2022 | Mosunetuzumab-axgb | GO29781 (NCT02500407) |
| 104 | 2022 | adenoviral vector-based gene therapy | Study CS-003 (NCT02773849) |
| 105 | 2022 | atezolizumab | Study ML39345 (NCT03141684) |
| 106 | 2022 | olutasidenib | Study 2102-HEM-101 (NCT02719574) |
| 107 | 2022 | mirvetuximab soravtansine-gynx | Study 0417 (NCT04296890) |
| 108 | 2022 | tremelimumab in combination with durvalumab and platinum-based chemotherapy | POSEIDON (NCT03164616) |
| 109 | 2022 | brentuximab vedotin in combination with chemotherapy | AHOD1331 (NCT02166463) |
| 110 | 2022 | cemiplimab-rwlc in combination with platinum-based chemotherapy | Study 16113 (NCT03409614) |
| 111 | 2022 | teclistamab-cqyv | MajesTEC-1 (NCT03145181) |
| 112 | 2022 | tremelimumab in combination with durvalumab | HIMALAYA (NCT03298451) |
| 113 | 2022 | futibatinib | TAS-120-101 (NCT02052778) |
| 114 | 2022 | selpercatinib | LIBRETTO-001 trial (NCT03157128) |
| 115 | 2022 | sodium thiosulfate | SIOPEL 6 (NCT00652132) |
| 116 | 2022 | sodium thiosulfate | COG ACCL0431 (NCT00716976) |
| 117 | 2022 | durvalumab | TOPAZ-1 (NCT03875235) |
| 118 | 2022 | fam-trastuzumab deruxtecan-nxki | DESTINY-Lung02 |
| 119 | 2022 | capmatinib | GEOMETRY mono-1 trial (NCT02414139) |
| 120 | 2022 | darolutamide tablets | ARASENS (NCT02799602) |
| 121 | 2022 | fam-trastuzumab deruxtecan-nxki | DESTINY-Breast04 (NCT03734029) |
| 122 | 2022 | crizotinib | A8081013 (NCT01121588) |
| 123 | 2022 | crizotinib | A8081013 (NCT01121588) |
| 124 | 2022 | crizotinib | ADVL0912 (NCT00939770) |
| 125 | 2022 | lisocabtagene maraleucel | TRANSFORM (NCT03575351) |
| 126 | 2022 | tisagenlecleucel | ELARA trial (NCT03568461) |
| 127 | 2022 | nivolumab in combination with fluoropyrimidine- and platinum-based chemotherapy | CHECKMATE-648 (NCT03143153) |
| 128 | 2022 | ivosidenib in combination with azacitidine | AG120-C-009, NCT03173248 |
| 129 | 2022 | azacitidine | AZA-JMML-001 (NCT02447666) |
| 130 | 2022 | fam-trastuzumab deruxtecan-nxki | DESTINY-Breast03 (NCT03529110) |
| 131 | 2022 | axicabtagene ciloleucel | ZUMA-7 |
| 132 | 2022 | lutetium Lu 177 vipivotide tetraxetan | VISION (NCT03511664) |
| 133 | 2022 | nivolumab and relatlimab-rmbw | RELATIVITY-047 (NCT03470922) |
| 134 | 2022 | olaparib | OlympiA (NCT02032823) |
| 135 | 2022 | nivolumab and platinum-doublet chemotherapy | CHECKMATE-816 (NCT02998528) |
| 136 | 2022 | ciltacabtagene autoleucel | CARTITUDE-1 (NCT03548207) |
| 137 | 2022 | tebentafusp-tebn | IMCgp100-202 (NCT03070392) |
| 138 | 2021 | pembrolizumab | KEYNOTE-716 (NCT03553836) |
| 139 | 2021 | rituximab plus chemotherapy | Inter-B-NHL Ritux 2010 (NCT01516580) |
| 140 | 2021 | daratumumab + hyaluronidase-fihj plus dexamethasone | PLEIADES (NCT03412565) |
| 141 | 2021 | pafolacianine | NCT03180307 |
| 142 | 2021 | pembrolizumab | KEYNOTE-564 (NCT03142334) |
| 143 | 2021 | asciminib | ASCEMBL (NCT03106779) |
| 144 | 2021 | asciminib | CABL001X2101 (NCT02081378) |
| 145 | 2021 | atezolizumab | IMpower010, NCT02486718 |
| 146 | 2021 | pembrolizumab in combination with chemotherapy | KEYNOTE-826 (NCT03635567) |
| 147 | 2021 | abemaciclib with endocrine therapy | monarchE (NCT03155997) |
| 148 | 2021 | brexucabtagene autoleucel | ZUMA-3 (NCT02614066) |
| 149 | 2021 | tisotumab vedotin-tftv | NCT03438396 |
| 150 | 2021 | cabozantinib | COSMIC-311 (NCT03690388) |
| 151 | 2021 | mobocertinib | Study 101 (NCT02716116) |
| 152 | 2021 | zanubrutinib | BGB-3111-214 (NCT03846427) |
| 153 | 2021 | zanubrutinib | ASPEN (NCT03053440) |
| 154 | 2022 | ivosidenib | Study AG120-C-005, NCT02989857 |
| 155 | 2022 | nivolumab | CHECKMATE-274 (NCT02632409) |
| 156 | 2022 | belzutifan | Study 004 (NCT03401788) |
| 157 | 2021 | lenvatinib plus pembrolizumab | CLEAR (Study 307/KEYNOTE-581; NCT02811861) |
| 158 | 2021 | pembrolizumab | KEYNOTE-522 (NCT03036488) |
| 159 | 2022 | pembrolizumab and lenvatinib | Study 309/KEYNOTE-775 (NCT03517449) |
| 160 | 2021 | daratumumab and hyaluronidase-fihj | APOLLO (NCT03180736) |
| 161 | 2021 | enfortumab vedotin-ejfv | EV-301 (NCT03474107) |
| 162 | 2021 | asparaginase erwinia chrysanthemi | Study JZP458-201 (NCT04145531) |
| 163 | 2021 | avapritinib | EXPLORER (NCT02561988) |
| 164 | 2021 | avapritinib | PATHFINDER (NCT03580655) |
| 165 | 2024 | infigratinib | CBGJ398X2204 (NCT02150967) |
| 166 | 2021 | sotorasib | CodeBreaK 100 (NCT03600883) |
| 167 | 2021 | amivantamab-vmjw | CHRYSALIS (NCT02609776) |
| 168 | 2021 | nivolumab | CHECKMATE-577 (NCT02743494) |
| 169 | 2021 | pembrolizumab | KEYNOTE-811 (NCT03615326) |
| 170 | 2021 | loncastuximab tesirine-lpyl | LOTIS-2 (NCT03589469) |
| 171 | 2021 | nivolumab in combination with chemotherapy | CHECKMATE-649 (NCT02872116) |
| 172 | 2024 | sacituzumab govitecan | TROPHY (IMMU-132-06; NCT03547973) |
| 173 | 2021 | sacituzumab govitecan | ASCENT; NCT02574455 |
| 174 | 2021 | isatuximab-irfc | IKEMA (NCT03275285) |
| 175 | 2021 | idecabtagene vicleucel | NCT03361748 |
| 176 | 2021 | pembrolizumab | KEYNOTE-590 (NCT03189719) |
| 177 | 2021 | tivozanib | TIVO-3 (NCT02627963) |
| 178 | 2021 | axicabtagene ciloleucel | ZUMA-5; NCT03105336 |
| 179 | 2021 | lorlatinib | Study B7461006 (NCT03052608) |
| 180 | 2024 | melphalan flufenamide | HORIZON (NCT02963493) |
| 181 | 2021 | cemiplimab-rwlc | Study 1624 (NCT03088540) |
| 182 | 2021 | cemiplimab-rwlc | Study 1620 (NCT03132636) |
| 183 | 2021 | lisocabtagene maraleucel | TRANSCEND (NCT02631044) |
| 184 | 2021 | umbralisib | UTX-TGR-205 (NCT02793583) |
| 185 | 2021 | tepotinib | VISION trial (NCT02864992) |
| 186 | 2021 | nivolumab plus cabozantinib | CHECKMATE-9ER (NCT03141177) |
| 187 | 2021 | fam-trastuzumab deruxtecan-nxki | DESTINY-Gastric01, NCT03329690 |
| 188 | 2020 | osimertinib | ADAURA, NCT02511106 |
| 189 | 2020 | relugolix | HERO (NCT03085095) |
| 190 | 2020 | selinexor | BOSTON Trial (KCP-330-023, NCT03110562) |
| 191 | 2020 | margetuximab | SOPHIA (NCT02492711) |
| 192 | 2020 | pralsetinib | ARROW, NCT03037385 |
| 193 | 2020 | naxitamab | Study 201 (NCT03363373) |
| 194 | 2020 | naxitamab | Study 12-230 (NCT01757626) |
| 195 | 2020 | pembrolizumab | KEYNOTE-355 (NCT02819518) |
| 196 | 2020 | venetoclax in combination | VIALE-A (NCT02993523) |
| 197 | 2020 | venetoclax in combination | VIALE-C (NCT03069352) |
| 198 | 2020 | pembrolizumab | KEYNOTE-204 (NCT02684292) |
| 199 | 2020 | nivolumab and ipilimumab | CHECKMATE-743 (NCT02899299) |
| 200 | 2020 | azacitidine tablets | QUAZAR (NCT01757535) |
| 201 | 2020 | carfilzomib and daratumumab | CANDOR (NCT03158688) |
| 202 | 2020 | carfilzomib and daratumumab | EQUULEUS (NCT01998971) |
| 203 | 2020 | belantamab mafodotin-blmf | DREAMM-2 (NCT03525678) |
| 204 | 2020 | tafasitamab-cxix | L-MIND (NCT02399085) |
| 205 | 2020 | atezolizumab | IMspire150, NCT02908672 |
| 206 | 2020 | brexucabtagene autoleucel | ZUMA-2 (NCT02601313) |
| 207 | 2020 | decitabine and cedazuridine | ASTX727-01-B (NCT02103478) |
| 208 | 2020 | decitabine and cedazuridine | ASTX727-02 (NCT03306264) |
| 209 | 2020 | avelumab | JAVELIN Bladder 100 trial (NCT02603432) |
| 210 | 2020 | pembrolizumab | KEYNOTE‑177 (NCT02563002) |
| 211 | 2020 | combination of pertuzumab, trastuzumab, and hyaluronidase-zzxf | FeDeriCa (NCT03493854) |
| 212 | 2020 | pembrolizumab | KEYNOTE-629 (NCT03284424) |
| 213 | 2020 | selinexor | KCP-330-009; NCT02227251 |
| 214 | 2020 | tazemetostat | two open-label, single-arm cohorts (Cohort 4 - EZH2 mutated FL and Cohort 5 - EZH2 wild-type FL) of a multi-center trial (Study E7438-G000-101, NCT01897571) |
| 215 | 2020 | gemtuzumab ozogamicin | AAML0531 (NCT00372593) |
| 216 | 2020 | nivolumab | ATTRACTION-3 (NCT02569242) |
| 217 | 2020 | ramucirumab plus erlotinib | RELAY (NCT02411448) |
| 218 | 2020 | atezolizumab plus bevacizumab | IMbrave150 (NCT03434379) |
| 219 | 2020 | nivolumab plus ipilimumab | CHECKMATE-9LA (NCT03215706) |
| 220 | 2020 | brigatinib | ALTA 1L (NCT02737501) |
| 221 | 2020 | olaparib | PROfound (NCT02987543) |
| 222 | 2020 | atezolizumab | IMpower110 (NCT02409342) |
| 223 | 2020 | ripretinib | INVICTUS (NCT03353753) |
| 224 | 2020 | rucaparib | TRITON2 (NCT02952534) |
| 225 | 2020 | nivolumab plus ipilimumab | CHECKMATE-227 (NCT02477826) |
| 226 | 2020 | pomalidomide | Study 12-C-0047 |
| 227 | 2020 | olaparib plus bevacizumab | PAOLA-1 (NCT03737643) |
| 228 | 2020 | selpercatinib | LIBRETTO-001 |
| 229 | 2020 | capmatinib | GEOMETRY mono-1 trial (NCT02414139) |
| 230 | 2020 | daratumumab and hyaluronidase-fihj | COLUMBA (NCT03277105) |
| 231 | 2020 | daratumumab and hyaluronidase-fihj | PLEIADES (NCT03412565) |
| 232 | 2020 | niraparib | PRIMA (NCT02655016) |
| 233 | 2020 | pembrolizumab | Study KEYNOTE-555 (NCT03665597) |
| 234 | 2020 | sacituzumab govitecan-hziy | IMMU-132-01 (NCT01631552) |
| 235 | 2020 | ibrutinib plus rituximab | E1912 trial (NCT02048813) |
| 236 | 2020 | pemigatinib | FIGHT-202 (NCT02924376) |
| 237 | 2020 | tucatinib | HER2CLIMB trial (NCT02614794) |
| 238 | 2020 | mitomycin | OLYMPUS (NCT02793128) |
| 239 | 2020 | selumetinib | SPRINT (NCT01362803) |
| 240 | 2020 | encorafenib in combination with cetuximab | BEACON CRC; NCT02928224 |
| 241 | 2020 | luspatercept-aamt | MEDALIST trial (NCT02631070) |
| 242 | 2020 | durvalumab | CASPIAN (NCT03043872)[排除中国] |
| 243 | 2020 | nivolumab and ipilimumab | CHECKMATE-040 (NCT01658878) |
| 244 | 2020 | isatuximab-irfc | ICARIA-MM (NCT02990338) |
| 245 | 2020 | neratinib | NALA (NCT01808573) |
| 246 | 2020 | tazemetostat | Cohort 5, Study EZH-202, NCT02601950 |
| 247 | 2020 | avapritinib | NAVIGATOR (NCT02508532) |
| 248 | 2019 | pembrolizumab | KEYNOTE-057 |
| 249 | 2019 | olaparib | POLO (NCT02184195) |
| 250 | 2019 | fam-trastuzumab deruxtecan-nxki | DESTINY-Breast01 (NCT03248492) |
| 251 | 2019 | enfortumab vedotin-ejfv | EV-201 (NCT03219333) |
| 252 | 2019 | enzalutamide | ARCHES (NCT02677896) |
| 253 | 2019 | atezolizumab with nab-paclitaxel and carboplatin | IMpower130 (NCT02367781) |
| 254 | 2019 | acalabrutinib | ELEVATE-TN (NCT02475681) |
| 255 | 2019 | acalabrutinib | ASCEND (NCT02970318) |
| 256 | 2019 | zanubrutinib | BGB-3111-206 (NCT03206970) |
| 257 | 2019 | zanubrutinib | BGB-3111-AU-003 (NCT02343120) |
| 258 | 2019 | niraparib | QUADRA (NCT02354586) |
| 259 | 2019 | daratumumab | CASSIOPEIA (NCT02541383) |
| 260 | 2019 | apalutamide | TITAN (NCT02489318) |
| 261 | 2019 | pembrolizumab | KEYNOTE‑181 (NCT02564263) |
| 262 | 2019 | pembrolizumab | KEYNOTE‑180 (NCT02559687) |
| 263 | 2019 | darolutamide | ARAMIS (NCT02200614) |
| 264 | 2019 | selinexor | Part 2 of STORM (KCP-330-012; NCT02336815) |
| 265 | 2019 | daratumumab | MAIA (NCT02252172) |
| 266 | 2019 | pembrolizumab | KEYNOTE-048 (NCT02358031) |
| 267 | 2019 | polatuzumab vedotin-piiq | Study GO29365 (NCT02257567) |
| 268 | 2019 | gilteritinib | ADMIRAL trial (NCT02421939) |
| 269 | 2019 | lenalidomide | AUGMENT (NCT01938001) |
| 270 | 2019 | alpelisib | SOLAR-1 (NCT02437318) |
| 271 | 2019 | venetoclax | CLL14 (NCT02242942) |
| 272 | 2019 | avelumab plus axitinib | JAVELIN Renal 101 (NCT02684006) |
| 273 | 2019 | ramucirumab | REACH‑2 (NCT02435433) |
| 274 | 2019 | ado-trastuzumab emtansine | KATHERINE (NCT01772472) |
| 275 | 2019 | ivosidenib | Study AG120-C-001, NCT02074839 |
| 276 | 2019 | pembrolizumab plus axitinib | KEYNOTE‑426 (NCT02853331) |
| 277 | 2019 | erdafitinib | Study BLC2001 (NCT02365597) |
| 278 | 2019 | pembrolizumab | KEYNOTE‑042 (NCT02220894) |
| 279 | 2019 | atezolizumab | IMpower133 (NCT02763579) |
| 280 | 2019 | atezolizumab | IMpassion130 (NCT02425891) |
| 281 | 2019 | trastuzumab and hyaluronidase-oysk | HannaH (NCT00950300) |
| 282 | 2019 | trastuzumab and hyaluronidase-oysk | SafeHER (NCT01566721) |
| 283 | 2019 | trifluridine/ tipiracil | TAGS (NCT02500043) |
| 284 | 2019 | pembrolizumab | EORTC1325/KEYNOTE‑054 (NCT02362594) |
| 285 | 2019 | cabozantinib | CELESTIAL (NCT01908426) |
| 286 | 2018 | tagraxofusp-erzs | STML-401-0114; NCT02113982 |
| 287 | 2018 | olaparib | SOLO-1 (NCT01844986) |
| 288 | 2018 | pembrolizumab | KEYNOTE-017 (NCT02267603) |
| 289 | 2018 | romiplostim | OAK (NCT02008227) |
| 290 | 2018 | romiplostim | NCT01903993 |
| 291 | 2018 | venetoclax | MURANO (NCT02005471) |
| 292 | 2018 | glasdegib | BRIGHT AML 1003, NCT01546038 |
| 293 | 2018 | emapalumab | NI-0501-04 (NCT01818492) |
| 294 | 2018 | brentuximab vedotin | ECHELON-2 (NCT01777152) |
| 295 | 2018 | pembrolizumab | KEYNOTE 224 (NCT02702414) |
| 296 | 2018 | lorlatinib | Study B7461001; NCT01970865 |
| 297 | 2018 | pembrolizumab | KEYNOTE-407 (NCT02775435) |
| 298 | 2018 | talazoparib | EMBRACA (NCT01945775) |
| 299 | 2018 | cemiplimab-rwlc | R2810-ONC-1540 |
| 300 | 2018 | dacomitinib | ARCHER 1050; NCT01774721 |
| 301 | 2018 | duvelisib | NCT02004522 |
| 302 | 2018 | moxetumomab pasudotox-tdfk | Study 1053 (NCT01829711) |
| 303 | 2018 | pembrolizumab in combination with chemotherapy | KEYNOTE-189 (NCT02578680) |
| 304 | 2018 | lenvatinib | REFLECT; NCT01761266 |
| 305 | 2018 | mogamulizumab-kpkc | Study 0761-010; NCT01728805 |
| 306 | 2018 | ribociclib | MONALEESA-7 (NCT02278120) |
| 307 | 2018 | ribociclib | MONALEESA-3 (NCT02422615) |
| 308 | 2018 | enzalutamide | PROSPER, NCT02003924 |
| 309 | 2018 | ipilimumab | Study CA209142 (CHECKMATE 142; NCT02060188) |
| 310 | 2018 | encorafenib and binimetinib | COLUMBUS; NCT01909453 |
| 311 | 2018 | pembrolizumab | KEYNOTE‑170 (NCT02576990) |
| 312 | 2018 | bevacizumab in combination with chemotherapy | GOG-0218 (NCT00262847) |
| 313 | 2018 | pembrolizumab | KEYNOTE 158 (NCT02628067) |
| 314 | 2018 | tisagenlecleucel | JULIET, NCT02445248 |
| 315 | 2018 | dabrafenib plus trametinib | COMBI-AD (NCT01682083) |
| 316 | 2018 | osimertinib | FLAURA, NCT02296125 |
| 317 | 2018 | nivolumab plus ipilimumab | CheckMate 214 (NCT02231749) |
| 318 | 2018 | rucaparib | ARIEL3 (NCT01968213) |
| 319 | 2018 | blinatumomab | BLAST trial (NCT01207388) |
| 320 | 2018 | nilotinib | CAMN107A2120 (NCT01077544) |
| 321 | 2018 | brentuximab vedotin | ECHELON-1 |
| 322 | 2018 | abemaciclib | MONARCH 3(NCT02246621) |
| 323 | 2018 | durvalumab after chemoradiation | PACIFIC (NCT02125461) |
| 324 | 2018 | apalutamide | SPARTAN, NCT01946204 |
| 325 | 2018 | abiraterone acetate in combination with prednisone | LATITUDE (NCT01715285) |
| 326 | 2018 | afatinib | LUX-Lung 2 [NCT00525148] |
| 327 | 2018 | afatinib | LUX-Lung 6 [NCT01121393] |
| 328 | 2018 | afatinib | LUX-Lung 3 [NCT00949650] |
| 329 | 2018 | olaparib | OlympiAD (NCT02000622) |

# eTable 2 Subgroup Categories and Definitions

| Subgroup Factor | Categories | Notes |
| --- | --- | --- |
| 1. Pediatric Trial Status | Yes, No | Excluded from analyzing age differences |
| 2. FDA Approval Year | 2018, 2019, 2020, 2021, 2022, 2023, 2024 | — |
| 3. Accelerated Approval | Yes, No | — |
| 4. Trial Phase | Phase 1, Phase 2, Phase 3 | — |
| 5. Trial Design | RCT, Non-RCT | — |
| 6. Cancer Type | Digestive System Cancers, Skin Cancers, Lung and Bronchus, Hematologic Malignancies, Breast Cancer, Endocrine Cancers, Respiratory System Cancers, Nervous System Cancers, Genitourinary Cancers, Childhood Cancers, Urinary System Cancers | — |
| 7. Trial Size | Small (<100), Intermediate (100–500), Large (>500) | Number of participants |
| 8. Industry Sponsorship | Yes, No | — |
| 9. Primary Endpoint | OS (Overall Survival), PFS/DFS/RFS/EFS, Others | — |
| 10. Sex Distribution | Male-dominated, Not male-dominated | Defined as male-dominated if male proportion > female; excluded from analyzing gender differences |

# eFigure 1 Sensitivity Meta-Analysis of Enrollment Incidence Ratios (EIRs)


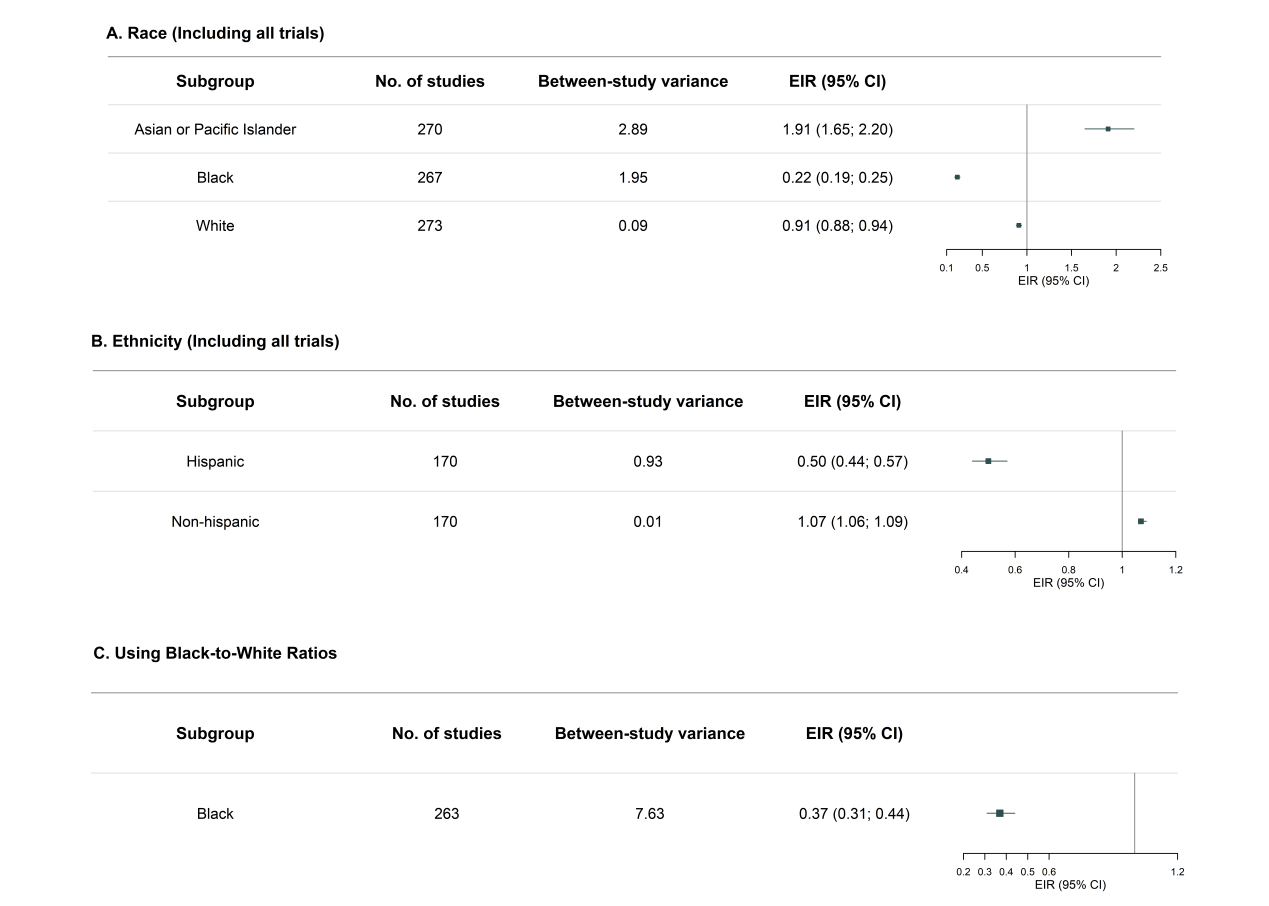


# eTable 3 Subgroup Analysis Results for Male Participant

| Subgroup | No. of studies | Between-study variance | EIR | 95% CI Lower Bound | 95% CI Upper Bound |
| --- | --- | --- | --- | --- | --- |
| 1. Pediatric Trial |  |  |  |  |  |
| Yes | 20 | 0.03 | 0.96 | 0.89 | 1.05 |
| No | 242 | 0.04 | 1.02 | 1 | 1.05 |
| 2. FDA Approval Year |  |  |  |  |  |
| 2018 | 32 | 0.06 | 1.01 | 0.93 | 1.09 |
| 2019 | 28 | 0.02 | 1.07 | 1.02 | 1.13 |
| 2020 | 49 | 0.03 | 1.03 | 0.98 | 1.08 |
| 2021 | 37 | 0.03 | 1.04 | 0.99 | 1.1 |
| 2022 | 31 | 0.06 | 1.01 | 0.92 | 1.1 |
| 2023 | 33 | 0.04 | 1.02 | 0.95 | 1.09 |
| 2024 | 52 | 0.05 | 0.97 | 0.91 | 1.02 |
| 3. Accelerated Approval |  |  |  |  |  |
| Yes | 57 | 0.04 | 0.97 | 0.92 | 1.02 |
| No | 205 | 0.04 | 1.03 | 1 | 1.06 |
| 4. Phase |  |  |  |  |  |
| Phase 1 | 16 | 0.01 | 0.99 | 0.93 | 1.05 |
| Phase 2 | 94 | 0.04 | 1 | 0.96 | 1.03 |
| Phase 3 | 152 | 0.04 | 1.03 | 1 | 1.07 |
| 5. RCT |  |  |  |  |  |
| Yes | 169 | 0.04 | 1.04 | 1 | 1.07 |
| No | 93 | 0.04 | 0.98 | 0.95 | 1.02 |
| 6. Disease |  |  |  |  |  |
| Genitourinary Cancers | 11 | 0 | 1.07 | 1.03 | 1.11 |
| Digestive System Cancers | 44 | 0.05 | 0.99 | 0.93 | 1.06 |
| Lung and Bronchus | 58 | 0.09 | 1.03 | 0.95 | 1.11 |
| Skin Cancers | 20 | 0.02 | 0.95 | 0.89 | 1.01 |
| Hematologic Malignancies | 90 | 0.02 | 1.03 | 1 | 1.06 |
| Endocrine Cancers | 4 | 0.05 | 1.16 | 0.94 | 1.44 |
| Respiratory System Cancers | 2 | 0 | 1.12 | 1.12 | 1.13 |
| Nervous System Cancers | 3 | 0.02 | 1.06 | 0.9 | 1.25 |
| Childhood Cancers | 17 | 0.03 | 0.93 | 0.85 | 1.02 |
| Urinary System Cancers | 11 | 0 | 1.08 | 1.05 | 1.11 |
| Head and Neck Cancers | 2 | 0 | 1.13 | 1.13 | 1.14 |
| 7. No. of participants |  |  |  |  |  |
| Small (<100) | 40 | 0.04 | 0.99 | 0.93 | 1.05 |
| Intermediate (100-500) | 144 | 0.04 | 0.99 | 0.96 | 1.02 |
| Large (>500) | 78 | 0.04 | 1.08 | 1.03 | 1.13 |
| 8. Industry Sponsored |  |  |  |  |  |
| Yes | 241 | 0.04 | 1.02 | 0.99 | 1.04 |
| No | 21 | 0.01 | 1.03 | 0.98 | 1.08 |
| 9. Primary Endpoint |  |  |  |  |  |
| OS | 60 | 0.03 | 1.13 | 1.08 | 1.18 |
| PFS/DFS/RFS/EFS | 68 | 0.04 | 0.98 | 0.94 | 1.03 |
| Others | 134 | 0.04 | 0.99 | 0.96 | 1.02 |

# eTable 4 Subgroup Analysis Results for White Participant

| Subgroup | No. of studies | Between-study variance | EIR | 95% CI Lower Bound | 95% CI Upper Bound |
| --- | --- | --- | --- | --- | --- |
| 1. Pediatric Trial |  |  |  |  |  |
| Yes | 12 | 0.06 | 0.91 | 0.8 | 1.05 |
| No | 196 | 0.04 | 0.99 | 0.96 | 1.02 |
| 2. FDA Approval Year |  |  |  |  |  |
| 2018 | 26 | 0.01 | 1.05 | 1 | 1.09 |
| 2019 | 23 | 0.02 | 1 | 0.94 | 1.06 |
| 2020 | 43 | 0.05 | 1.01 | 0.94 | 1.08 |
| 2021 | 24 | 0.03 | 1.03 | 0.97 | 1.1 |
| 2022 | 26 | 0.09 | 0.9 | 0.81 | 1.01 |
| 2023 | 28 | 0.03 | 1 | 0.94 | 1.06 |
| 2024 | 38 | 0.06 | 0.93 | 0.86 | 1.01 |
| 3. Accelerated Approval |  |  |  |  |  |
| Yes | 44 | 0.03 | 1.02 | 0.97 | 1.07 |
| No | 164 | 0.05 | 0.98 | 0.95 | 1.01 |
| 4. Phase |  |  |  |  |  |
| Phase 1 | 7 | 0.01 | 1.06 | 0.99 | 1.13 |
| Phase 2 | 64 | 0.05 | 1.01 | 0.95 | 1.06 |
| Phase 3 | 137 | 0.04 | 0.97 | 0.94 | 1.01 |
| 5. RCT |  |  |  |  |  |
| Yes | 147 | 0.04 | 0.97 | 0.94 | 1.01 |
| No | 61 | 0.05 | 1.02 | 0.97 | 1.08 |
| 6. Disease |  |  |  |  |  |
| Digestive System Cancers | 25 | 0.11 | 0.95 | 0.83 | 1.08 |
| Skin Cancers | 16 | 0.01 | 1.01 | 0.96 | 1.07 |
| Hematologic Malignancies | 73 | 0.03 | 1.04 | 1 | 1.09 |
| Breast Cancer | 25 | 0.06 | 0.9 | 0.82 | 0.99 |
| Endocrine Cancers | 4 | 0.03 | 0.95 | 0.81 | 1.11 |
| Respiratory System Cancers | 2 | 0.01 | 1.05 | 0.94 | 1.18 |
| Nervous System Cancers | 2 | 0.27 | 0.65 | 0.33 | 1.27 |
| Genitourinary Cancers | 39 | 0.02 | 1.02 | 0.97 | 1.07 |
| Childhood Cancers | 11 | 0.01 | 1.01 | 0.95 | 1.07 |
| Urinary System Cancers | 11 | 0.02 | 0.93 | 0.85 | 1.01 |
| 7. No. of participants |  |  |  |  |  |
| Small (<100) | 26 | 0.06 | 1.01 | 0.92 | 1.11 |
| Intermediate (100-500) | 107 | 0.03 | 1 | 0.97 | 1.04 |
| Large (>500) | 75 | 0.05 | 0.95 | 0.91 | 1 |
| 8. Industry Sponsored |  |  |  |  |  |
| Yes | 192 | 0.04 | 0.98 | 0.96 | 1.01 |
| No | 16 | 0.06 | 1.02 | 0.9 | 1.15 |
| 9. Primary Endpoint |  |  |  |  |  |
| OS | 38 | 0.06 | 0.91 | 0.84 | 0.98 |
| PFS/DFS/RFS/EFS | 75 | 0.04 | 1 | 0.95 | 1.04 |
| Others | 95 | 0.04 | 1.01 | 0.97 | 1.05 |
| 10. Male Dominated Trial |  |  |  |  |  |
| Yes | 156 | 0.03 | 1.02 | 0.99 | 1.05 |
| No | 52 | 0.06 | 0.88 | 0.82 | 0.94 |

# eTable 5 Subgroup Analysis Results for Asian or Pacific Islander individuals

| Subgroup | No. of studies | Between-study variance | EIR | 95% CI Lower Bound | 95% CI Upper Bound |
| --- | --- | --- | --- | --- | --- |
| 1. Pediatric Trial |  |  |  |  |  |
| Yes | 12 | 1.65 | 1.14 | 0.63 | 2.07 |
| No | 191 | 2.51 | 1.53 | 1.3 | 1.8 |
| 2. FDA Approval Year |  |  |  |  |  |
| 2018 | 24 | 2.09 | 1.23 | 0.78 | 1.95 |
| 2019 | 23 | 2.13 | 2.18 | 1.39 | 3.4 |
| 2020 | 42 | 1.65 | 1.46 | 1.07 | 2 |
| 2021 | 23 | 2.24 | 1.44 | 0.92 | 2.25 |
| 2022 | 26 | 3.04 | 1.4 | 0.87 | 2.25 |
| 2023 | 27 | 3.25 | 1.32 | 0.83 | 2.11 |
| 2024 | 38 | 3.66 | 1.63 | 1.09 | 2.42 |
| 3. Accelerated Approval |  |  |  |  |  |
| Yes | 45 | 2.79 | 1.3 | 0.92 | 1.84 |
| No | 158 | 2.37 | 1.57 | 1.31 | 1.87 |
| 4. Phase |  |  |  |  |  |
| Phase 1 | 6 | 2.21 | 1.38 | 0.57 | 3.33 |
| Phase 2 | 64 | 2.27 | 1.19 | 0.9 | 1.58 |
| Phase 3 | 133 | 2.49 | 1.68 | 1.38 | 2.04 |
| 5. RCT |  |  |  |  |  |
| Yes | 144 | 2.4 | 1.63 | 1.35 | 1.96 |
| No | 59 | 2.47 | 1.22 | 0.91 | 1.65 |
| 6. Disease |  |  |  |  |  |
| Digestive System Cancers | 25 | 4.96 | 1.23 | 0.7 | 2.16 |
| Skin Cancers | 15 | 0.74 | 1.14 | 0.71 | 1.84 |
| Hematologic Malignancies | 70 | 2.75 | 1.18 | 0.89 | 1.56 |
| Breast Cancer | 25 | 1.06 | 2.41 | 1.73 | 3.36 |
| Endocrine Cancers | 4 | 1.71 | 1.46 | 0.55 | 3.88 |
| Respiratory System Cancers | 2 | 143.26 | 0.47 | 0.02 | 10.48 |
| Nervous System Cancers | 2 | 10.88 | 2.48 | 0.28 | 21.95 |
| Genitourinary Cancers | 38 | 2.16 | 1.6 | 1.14 | 2.26 |
| Childhood Cancers | 11 | 1.02 | 1.08 | 0.64 | 1.84 |
| Urinary System Cancers | 11 | 1.09 | 3.21 | 1.93 | 5.35 |
| 7. No. of participants |  |  |  |  |  |
| Small (<100) | 26 | 2.27 | 1.55 | 0.99 | 2.41 |
| Intermediate (100-500) | 102 | 2.38 | 1.25 | 1 | 1.57 |
| Large (>500) | 75 | 2.42 | 1.89 | 1.46 | 2.44 |
| 8. Industry Sponsored |  |  |  |  |  |
| Yes | 187 | 2.28 | 1.62 | 1.38 | 1.9 |
| No | 16 | 1.65 | 0.62 | 0.37 | 1.03 |
| 9. Primary Endpoint |  |  |  |  |  |
| OS | 38 | 3.6 | 2.21 | 1.48 | 3.29 |
| PFS/DFS/RFS/EFS | 71 | 1.78 | 1.63 | 1.28 | 2.08 |
| Others | 94 | 2.32 | 1.2 | 0.95 | 1.51 |
| 10. Male Dominated Trial |  |  |  |  |  |
| Yes | 153 | 2.86 | 1.28 | 1.05 | 1.55 |
| No | 50 | 0.97 | 2.38 | 1.89 | 3 |

# eTable 6 Subgroup Analysis Results for non-Hispanic individuals

| Subgroup | No. of studies | Between-study variance | EIR | 95% CI Lower Bound | 95% CI Upper Bound |
| --- | --- | --- | --- | --- | --- |
| 1. Pediatric Trial |  |  |  |  |  |
| Yes | 6 | 0.01 | 1.12 | 1.02 | 1.22 |
| No | 124 | 0.01 | 1.08 | 1.06 | 1.1 |
| 2. FDA Approval Year |  |  |  |  |  |
| 2018 | 12 | 0.01 | 1.14 | 1.06 | 1.21 |
| 2019 | 17 | 0 | 1.08 | 1.05 | 1.12 |
| 2020 | 29 | 0.01 | 1.07 | 1.04 | 1.1 |
| 2021 | 19 | 0.01 | 1.03 | 0.98 | 1.08 |
| 2022 | 14 | 0.01 | 1.08 | 1.04 | 1.13 |
| 2023 | 16 | 0.02 | 1.08 | 1.02 | 1.16 |
| 2024 | 23 | 0.02 | 1.09 | 1.02 | 1.16 |
| 3. Accelerated Approval |  |  |  |  |  |
| Yes | 25 | 0.01 | 1.14 | 1.1 | 1.19 |
| No | 105 | 0.01 | 1.06 | 1.04 | 1.09 |
| 4. Phase |  |  |  |  |  |
| Phase 1 | 4 | 0 | 1.02 | 0.96 | 1.09 |
| Phase 2 | 33 | 0.01 | 1.11 | 1.08 | 1.14 |
| Phase 3 | 93 | 0.01 | 1.07 | 1.04 | 1.1 |
| 5. RCT |  |  |  |  |  |
| Yes | 95 | 0.01 | 1.07 | 1.05 | 1.1 |
| No | 35 | 0.01 | 1.1 | 1.06 | 1.13 |
| 6. Disease |  |  |  |  |  |
| Genitourinary Cancers | 25 | 0.02 | 1.04 | 0.99 | 1.11 |
| Skin Cancers | 12 | 0.01 | 1.03 | 0.98 | 1.08 |
| Digestive System Cancers | 17 | 0 | 1.15 | 1.11 | 1.19 |
| Hematologic Malignancies | 46 | 0.01 | 1.11 | 1.07 | 1.15 |
| Breast Cancer | 16 | 0 | 1.03 | 1 | 1.06 |
| Endocrine Cancers | 2 | 0.01 | 1.04 | 0.92 | 1.17 |
| Childhood Cancers | 6 | 0.01 | 1.12 | 1.02 | 1.22 |
| Urinary System Cancers | 5 | 0 | 1.04 | 1 | 1.09 |
| 7. No. of participants |  |  |  |  |  |
| Small (<100) | 14 | 0.01 | 1.09 | 1.02 | 1.15 |
| Intermediate (100-500) | 64 | 0.01 | 1.1 | 1.07 | 1.13 |
| Large (>500) | 52 | 0.01 | 1.06 | 1.03 | 1.09 |
| 8. Industry Sponsored |  |  |  |  |  |
| Yes | 118 | 0.01 | 1.07 | 1.05 | 1.1 |
| No | 12 | 0.01 | 1.12 | 1.06 | 1.2 |
| 9. Primary Endpoint |  |  |  |  |  |
| OS | 26 | 0.01 | 1.07 | 1.03 | 1.12 |
| PFS/DFS/RFS/EFS | 47 | 0.01 | 1.06 | 1.03 | 1.08 |
| Others | 57 | 0.02 | 1.1 | 1.06 | 1.14 |
| 10. Male Dominated Trial |  |  |  |  |  |
| Yes | 99 | 0.01 | 1.08 | 1.05 | 1.11 |
| No | 31 | 0.01 | 1.07 | 1.05 | 1.11 |

# eTable 7 Subgroup Analysis Results for younger adults

| Subgroup | No. of studies | Between-study variance | EIR | 95% CI Lower Bound | 95% CI Upper Bound |
| --- | --- | --- | --- | --- | --- |
| 1. FDA Approval Year |  |  |  |  |  |
| 2018 | 19 | 1.03 | 1.18 | 0.81 | 1.72 |
| 2019 | 22 | 1.49 | 1.12 | 0.75 | 1.67 |
| 2020 | 31 | 0.21 | 1.17 | 1.01 | 1.37 |
| 2021 | 23 | 0.1 | 1.3 | 1.14 | 1.47 |
| 2022 | 17 | 0.09 | 1.51 | 1.31 | 1.74 |
| 2023 | 13 | 0.44 | 1.2 | 0.86 | 1.66 |
| 2024 | 25 | 0.47 | 1.37 | 1.07 | 1.75 |
| 2. Accelerated Approval |  |  |  |  |  |
| Yes | 28 | 0.16 | 1.38 | 1.2 | 1.6 |
| No | 122 | 0.53 | 1.23 | 1.09 | 1.38 |
| 3. Phase |  |  |  |  |  |
| Phase 1 | 6 | 0.22 | 1.47 | 1.03 | 2.09 |
| Phase 2 | 42 | 0.44 | 1.27 | 1.06 | 1.52 |
| Phase 3 | 102 | 0.48 | 1.24 | 1.09 | 1.39 |
| 4. RCT |  |  |  |  |  |
| Yes | 110 | 0.44 | 1.22 | 1.09 | 1.37 |
| No | 40 | 0.47 | 1.34 | 1.1 | 1.63 |
| 5. Disease |  |  |  |  |  |
| Lung and Bronchus | 30 | 0.1 | 1.81 | 1.62 | 2.02 |
| Skin Cancers | 8 | 0.28 | 1.34 | 0.95 | 1.89 |
| Hematologic Malignancies | 44 | 1.48 | 0.89 | 0.67 | 1.18 |
| Endocrine Cancers | 2 | 0.09 | 1.4 | 0.93 | 2.12 |
| Respiratory System Cancers | 2 | 0.01 | 1.11 | 0.95 | 1.31 |
| Genitourinary Cancers | 19 | 0.04 | 1.17 | 1.07 | 1.29 |
| Digestive System Cancers | 25 | 0.04 | 1.32 | 1.21 | 1.43 |
| Urinary System Cancers | 5 | 0.06 | 1.44 | 1.15 | 1.79 |
| Breast Cancer | 13 | 0.02 | 1.52 | 1.41 | 1.64 |
| 6. No. of participants |  |  |  |  |  |
| Small (<100) | 11 | 0.18 | 1.46 | 1.15 | 1.87 |
| Intermediate (100-500) | 81 | 0.43 | 1.21 | 1.06 | 1.37 |
| Large (>500) | 58 | 0.54 | 1.28 | 1.08 | 1.52 |
| 7. Industry Sponsored |  |  |  |  |  |
| Yes | 146 | 0.46 | 1.25 | 1.13 | 1.38 |
| No | 4 | 0.09 | 1.31 | 0.98 | 1.76 |
| 8. Male Dominated Trial |  |  |  |  |  |
| Yes | 118 | 0.55 | 1.16 | 1.03 | 1.31 |
| No | 32 | 0.04 | 1.65 | 1.53 | 1.77 |
| 9. Primary Endpoint |  |  |  |  |  |
| OS | 43 | 0.29 | 1.26 | 1.08 | 1.46 |
| PFS/DFS/RFS/EFS | 53 | 0.74 | 1.29 | 1.05 | 1.57 |
| Others | 54 | 0.36 | 1.22 | 1.05 | 1.41 |

# eTable 8 Results of multivariable meta-regression

| Variable | Age 65+ | | Hispanic | | Female | | Black | |
| --- | --- | --- | --- | --- | --- | --- | --- | --- |
|  | Regression coefficient (95% CI) | p value | Regression coefficient (95% CI) | p value | Regression coefficient (95% CI) | p value | Regression coefficient (95% CI) | p value |
| Phase (3 VS 2 VS 1) | 0.052 (-0.264, 0.367) | 0.749 | -0.237 (-0.66, 0.187) | 0.273 | 0.048 (-0.065, 0.16) | 0.406 | -0.677 (-1.127, -0.227) | 0.003 |
| RCT (Yes VS NO) | 0.029 (-0.356, 0.413) | 0.884 | 0.049 (-0.449, 0.547) | 0.847 | -0.039 (-0.174, 0.097) | 0.577 | 0.313 (-0.191, 0.816) | 0.223 |
| Sample (>500 VS 100-500 VS <100) | 0.111 (-0.094, 0.316) | 0.289 | 0.068 (-0.201, 0.336) | 0.622 | -0.023 (-0.103, 0.056) | 0.565 | -0.088 (-0.341, 0.165) | 0.494 |
| Male.dominated (Yes VS NO) | 0.483 (0.247, 0.718) | <0.001 | -0.092 (-0.389, 0.205) | 0.545 | / | / | -0.032 (-0.319, 0.254) | 0.824 |
| Approval.Year | -0.017 (-0.065, 0.031) | 0.491 | 0.045 (-0.022, 0.111) | 0.191 | 0.014 (-0.005, 0.032) | 0.148 | 0.025 (-0.038, 0.088) | 0.437 |
| Accelerated.approval(Yes VS NO) | 0.006 (-0.287, 0.299) | 0.968 | -0.548 (-0.929, -0.167) | 0.005 | -0.001 (-0.104, 0.101) | 0.978 | -0.346 (-0.71, 0.018) | 0.063 |
| Endpoint (Others VS PFS/DFS/RFS/EFS VS OS) | 0.093 (-0.096, 0.282) | 0.337 | -0.157 (-0.412, 0.098) | 0.228 | 0.108 (0.036, 0.179) | 0.003 | 0.281 (0.022, 0.54) | 0.034 |
| Industry.sponsored (Yes VS NO) | 0.095 (-0.498, 0.687) | 0.754 | 0.115 (-0.427, 0.658) | 0.677 | 0.025 (-0.135, 0.184) | 0.763 | -0.642 (-1.15, -0.133) | 0.013 |
| Pediatric.trial (Yes VS NO) | / | / | 0.258 (-0.471, 0.987) | 0.488 | 0.111 (-0.055, 0.278) | 0.19 | 0.899 (0.329, 1.47) | 0.002 |
| Survival of indication (Increase of 20% in 5-year survival) | 0.031 (-0.043, 0.105) | 0.411 | 0.071 (-0.035, 0.176) | 0.189 | -0.023 (-0.051, 0.004) | 0.097 | 0.036 (-0.067, 0.139) | 0.496 |

^a^ The regression coefficient represents the variation in the logarithmic enrollment-incidence ratio (logEIR) for a specific trial category in comparison to the reference category. For instance, in trials with an OS primary endpoint, the logEIR for Hispanic patients increases by 0.95 units relative to trials with a non-OS primary endpoint.

^b^ OS, overall survival

# eTable 9 Multicollinearity Analysis Results

| Variable | VIF | | | |
| --- | --- | --- | --- | --- |
|  | Age 65+ | Hispanic | Black | Female |
| Phase | 3.833 | 2.911 | 3.416 | 3.429 |
| RCT | 3.649 | 3.101 | 2.905 | 3.087 |
| Sample | 1.888 | 1.898 | 1.838 | 1.998 |
| Male-dominated | 1.404 | 1.041 | 1.036 | / |
| Approval Year | 2.525 | 1.078 | 1.038 | 1.054 |
| Accelerated Approval | 1.637 | 1.369 | 1.39 | 1.319 |
| Endpoint | 3.067 | 2.422 | 2.448 | 2.494 |
| Industry-sponsored | 1.159 | 1.607 | 1.236 | 1.315 |
| Pediatric | / | 1.528 | 1.278 | 1.387 |
| Survival | 1.452 | 1.367 | 1.273 | 1.198 |

# eTable 10 Study-level EIRs: Pandemic (enrollment midpoint 2020-2022) vs non-pandemic

| Subgroup | Pandemic n | Pandemic EIR median (IQR) | Non-pandemic n | Non-pandemic EIR median (IQR) | Median difference (P minus NP) | P value |
| --- | --- | --- | --- | --- | --- | --- |
| Age 65+ | 49 | 0.6445 (0.4966 to 0.8203) | 101 | 0.7625 (0.5802 to 0.9190) | -0.118 | 0.05392 |
| Age under 65 | 49 | 1.4670 (1.1531 to 1.8946) | 101 | 1.3671 (1.1277 to 1.6043) | 0.0999 | 0.1072 |
| Hispanic | 40 | 0.4192 (0.2893 to 0.7197) | 90 | 0.5270 (0.2765 to 0.9044) | -0.1078 | 0.6372 |
| Non-Hispanic | 40 | 1.1109 (1.0575 to 1.1376) | 90 | 1.0691 (1.0199 to 1.1338) | 0.0418 | 0.2815 |
| Male | 101 | 1.0032 (0.9275 to 1.1224) | 161 | 1.0540 (0.9299 to 1.1339) | -0.0508 | 0.2928 |
| Female | 101 | 0.9957 (0.8246 to 1.0914) | 160 | 0.9275 (0.7429 to 1.0884) | 0.0682 | 0.1928 |
| Asian | 72 | 1.4472 (0.5575 to 3.8550) | 131 | 1.7416 (0.4793 to 3.4809) | -0.2944 | 0.5666 |
| Black or African | 71 | 0.2834 (0.1087 to 0.5483) | 131 | 0.1938 (0.1033 to 0.4132) | 0.0896 | 0.2555 |
| White | 73 | 1.0034 (0.8411 to 1.1068) | 135 | 1.0462 (0.9465 to 1.1331) | -0.0428 | 0.02432 |

# Figure S1. Study selection flow diagram


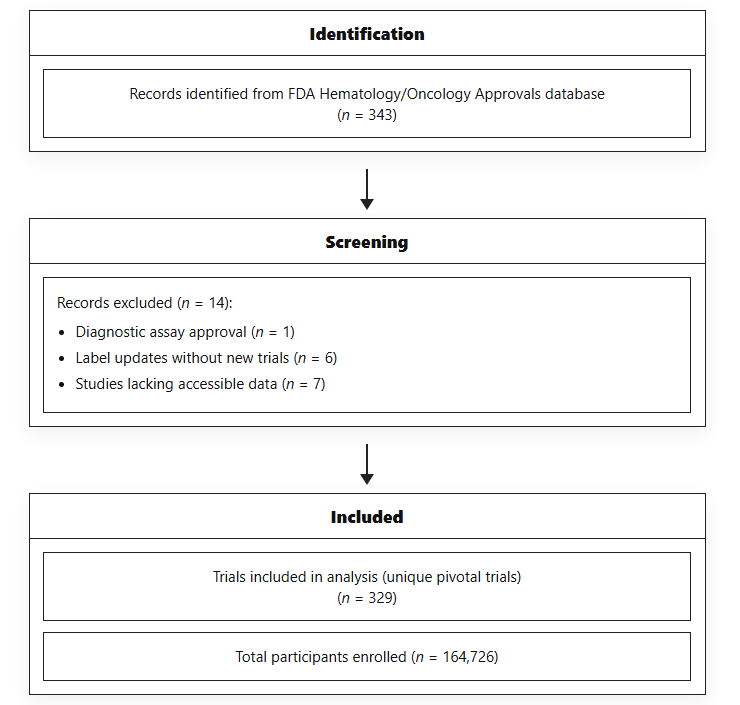


Reference

[1] Sweeting M J, Sutton A J, Lambert P C. What to add to nothing? Use and avoidance of continuity corrections in meta-analysis of sparse data[J]. Stat Med, 2004,23(9):1351-1375.

[2] Bradburn M J, Deeks J J, Berlin J A, et al. Much ado about nothing: a comparison of the performance of meta-analytical methods with rare events[J]. Stat Med, 2007,26(1):53-77.

[3] Riaz I B, Islam M, Ikram W, et al. Disparities in the Inclusion of Racial and Ethnic Minority Groups and Older Adults in Prostate Cancer Clinical Trials: A Meta-analysis[J]. JAMA Oncol, 2023,9(2):180-187.

[4] Loree J M, Anand S, Dasari A, et al. Disparity of Race Reporting and Representation in Clinical Trials Leading to Cancer Drug Approvals From 2008 to 2018[J]. JAMA Oncol, 2019,5(10):e191870.

[5] Owens-Walton J, Williams C, Rompré-Brodeur A, et al. Minority Enrollment in Phase II and III Clinical Trials in Urologic Oncology[J]. J Clin Oncol, 2022,40(14):1583-1589.

[6] Riaz I B, Islam M, Khan A M, et al. Disparities in Representation of Women, Older Adults, and Racial/Ethnic Minorities in Immune Checkpoint Inhibitor Trials[J]. Am J Med, 2022,135(8):984-992.

[7] Kim E S, Bruinooge S S, Roberts S, et al. Broadening eligibility criteria to make clinical trials more representative: American Society of Clinical Oncology and Friends of Cancer Research Joint Research Statement[J]. Journal of Clinical Oncology, 2017, 35(33): 3737-3744.

[8] Fern L A, Whelan J S. Recruitment of adolescents and young adults to cancer clinical trials—international comparisons, barriers, and implications[C]//Seminars in oncology. WB Saunders, 2010, 37(2): e1-e8.

[9] Beaver J A, Howie L J, Pelosof L, et al. A 25-year experience of US Food and Drug Administration accelerated approval of malignant hematology and oncology drugs and biologics: a review[J]. JAMA oncology, 2018, 4(6): 849-856.

[10] Unger J M, Vaidya R, Hershman D L, et al. Systematic review and meta-analysis of the magnitude of structural, clinical, and physician and patient barriers to cancer clinical trial participation[J]. JNCI: Journal of the National Cancer Institute, 2019, 111(3): 245-255.
